# Dual‐Site Synergistic Mechanism via Single‐Atom and Vacancy Drives Lattice Oxygen Activation in Layered Double Hydroxides

**DOI:** 10.1002/advs.202515407

**Published:** 2025-12-05

**Authors:** Shixin Wu, Wenyu Lu, Shijun Zhao, Kai Zhao, Ning Yan, Liqiu Huang, Derun Li, Tao Jiang, Hengyi Wu, Feng Ren

**Affiliations:** ^1^ School of Physics and Technology, and Hubei Nuclear Solid Physics Key Laboratory Wuhan University Wuhan 430072 China; ^2^ Department of Mechanical Engineering City University of Hong Kong Hong Kong China; ^3^ Center for Ion Beam Application Center for Electron Microscopy and MOE Key Laboratory of Artificial Micro‐ and Nano‐Structures Wuhan University Wuhan 430072 China

**Keywords:** alkaline water electrocatalyst, ion beam technology, LOM pathway, single atoms and vacancy, synergistic regulation

## Abstract

Establishing the synergistic effect between single atoms and vacancies for the lattice oxygen mechanism (LOM) pathway of the oxygen evolution reaction (OER) is crucial for developing robust and efficient catalysts, yet remains unexplored. Herein, an ion irradiation‐assisted strategy is first applied to introduce controlled oxygen vacancies into NiFe layer double hydroxides (LDH), enabling the firm anchoring of Mo single atoms with a high loading of 7.4 wt.%. The precise regulation facilitates synergistic activation of lattice oxygen via Mo atoms and vacancies. Thus, the optimized ^SA^Mo‐NiFe LDH/Ti delivers remarkably improved performance with a decrease of overpotential of 226 mV at 10 mA cm^−2^ and, owing to the confinement effect of vacancies, maintains 600 h at 500 mA cm^−2^. First‐principles calculations reveal that Mo single atoms coupling with oxygen vacancies exhibit enhanced adsorption capability, and promote lattice oxygen activation, synergistically optimizing the electronic structure of active centers for OER. This study establishes a direct link between defect engineering, single‐atom catalysis, and LOM, providing a robust strategy for rational catalyst design.

## Graphical abstract

A strategy utilizing high‐energy ion beam technology to engineer defects in layered double hydroxide (LDH) nanosheets enables the high loading of non‐noble metal Mo single atoms. The synergistic effect between oxygen vacancies and single atoms promotes a novel mechanism for lattice oxygen activation, thereby significantly enhancing the oxygen evolution reaction (OER) performance.

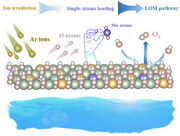

## 1 Introduction

Electrochemical water‐splitting has been the future trend of hydrogen production, yet the half reactions (OER) during water splitting, consisting of a four‐proton to‐electron‐transfer reaction process (4OH^−^ → 2H_2_O + O_2_ + 4e^−^ in base), cause the sluggish kinetics and a constraint in the process of water splitting. Therefore, designing efficient non‐noble metal catalysts based on the earth‐abundant elements (such as Ni, Fe) for OER is essential to achieve higher efficiency in water splitting as well as large‐scale industrial hydrogen production. Transition metal‐based layer double hydroxides are widely regarded as one of the most efficient and promising electrocatalysts due to their economically feasible formulation, variability of the interlayer anions, unique layered two‐dimensional structure, and intrinsic electronic distribution.^[^ ^1^, ^2^, ^3^, ^4^ ^]^ NiFe‐based double‐layer hydroxides have been favored by numerous researchers for their superior OER performance.^[^ ^5^, ^6^ ^]^ For example, Gong et al. first reported the use of highly crystalline NiFe LDH for efficient oxygen evolution, demonstrating its exceptionally high electrocatalytic activity.^[^ ^7^ ^]^Although transition metal atoms in layered double hydroxides (LDHs) are typically regarded as active sites for the oxygen evolution reaction (OER), a significant number of inert oxygen atoms are also present on the surface. These inert oxygen atoms do not participate in the catalytic process and conversely impede the transport of electrons, thereby limiting the overall catalytic performance of LDHs.^[^ ^8^, ^9^ ^]^

To maximally exploit the surface sites and improve the OER performance of transition metal hydroxides, it is critical, but challengeable, to regulate the surface metal atoms while simultaneously activating the catalytic role of oxygen atoms. Therefore, some studies have been carried out to optimize the OER performance of LDH by constructing pore structures,^[^ ^10^ ^]^ building heterogeneous structures,^[^ ^11^ ^]^ doping metal ions,^[^ ^12^ ^]^ introducing defects and other strategies.^[^ ^13^, ^14^ ^]^ For example, the Shao group systematically investigated the performance of NiFe LDH on iron foam with regulated morphologies via corrosion engineering, and achieved optimal catalytic activity.^[^ ^15^ ^]^ Nevertheless, how to utilize defect engineering strategy to optimize LDH activity is considered as a way to develop more appropriate and more efficient OER catalysts from a perspective of intrinsic mechanism.^[^ ^16^, ^17^, ^18^ ^]^ Some researchers have demonstrated the existence of synergistic coupling of different defect sites in LDH, including anionic and cationic vacancies.^[^ ^19^, ^20^, ^21^ ^]^ The introduction of defect sites can activate the electroactivity of both the metal and oxygen sites, causing a redistribution of the electronic states on the surface of catalyst and building a ligand environment change at its interface, which can enhance electrical conductivity, accelerate the charge transfer in reaction, and enrich the active centers. For example, Wang et al. synthesized NiFe LDH nanosheets with abundant metal and oxygen vacancies on the surface of Nickel foam using electron‐withdrawing organic molecules.^[^ ^22^ ^]^ Although the stability could only be maintained at low density current of 50 mA cm^−2^, these nanosheets exhibited a low overpotential of 230 mV at a current density of 100 mA cm^−^^2^ and a Tafel slope of 37.1 mV dec^−1^, showcasing decent OER performance. In addition, the defect sites can provide unique anchors for trapping other metal species as doped metal atoms or even single atoms to activate electrochemical reactions.

Single‐atom catalysts have excellent catalytic activity due to their unique electronic structure and maximum atomic utilization.^[^ ^23^, ^24^, ^25^, ^26^ ^]^ However, it faces limitations of low loading, poor stability, and the use of rare noble metals. The existence of unsaturated coordination bonds induced by abundant defects, which can interact with single atoms and exert confinement effects, is reasonably considered as a potential strategy to address the inherent limitations of single‐atom catalysts, particularly their low loading and poor stability. Therefore, how to anchor non‐noble metal single atoms with high loading on NiFe LDH by defect engineering is the key to prepare efficient and stable LDH catalysts. Notably, there are some strategies for generating defects on the surface of layered double hydroxides, yet it is difficult to control the appropriate number of vacancies.^[^ ^27^, ^28^ ^]^

To date, it is widely accepted that single‐atom and defect‐engineered catalysts for oxygen evolution reaction (OER) predominantly operate via the adsorbate evolution mechanism (AEM) and the lattice oxygen mechanism (LOM), and many studies have demonstrated that heteroatoms or defects can significantly promote the LOM and thereby enhance OER electrocatalytic performance.^[^ ^29^, ^30^, ^31^, ^32^ ^]^ Nevertheless, the concomitant atoms dissolution associated with the LOM pathway severely compromises the long‐term stability of the catalysts, thus imposing significant limitations on their potential for commercial application. Given the potential of vacancies to stabilize single‐atom migration and suppress dissolution, and considering the current lack of understanding on the synergistic impact of vacancies and single atoms on LOM‐related catalytic behavior, it is of paramount importance to establish the correlation between single atoms and vacancies at the atomic level and explore their synergistic effects.

In this work, we first report an ion irradiation‐assisted strategy to fabricate a controlled number of oxygen vacancies for anchoring Mo single atoms on the surface of NiFe LDH as an efficient and stable OER electrocatalyst. Ion beam technology is recognized to enable precise modulation of vacancy in catalytic materials due to its accuracy, repeatability, and controllability.^[^ ^33^, ^34^, ^35^, ^36^ ^]^ The single‐atomic‐site Mo catalyst stabilized on the NiFe LDH nanosheets were prepared through anchoring of Mo single atoms by the ion‐irradiation‐introduced vacancies and subsequent simple hydrothermal procedure. Combined with spherical aberration‐corrected transmission electron microscopy as well as X‐ray absorption fine‐structure spectroscopy, X‐ray photoelectron spectroscopy analyses, it was proved that this strategy successfully achieves loading of non‐noble metal single atoms through vacancy regulation is revealed. The best synthesized ^SA^Mo‐NiFe LDH/Ti reaches a high Mo loading of 7.4 wt.%. Moreover, the sample achieves a remarkable reduction in overpotential by 226 mV at 10 mA cm^−2^ than that of the pristine NiFe LDH nanosheets, greatly enhancing OER activity. In addition, the domain‐limiting effect of vacancies effectively stabilizes the migration of single atoms and prevents their loss, thus, the best sample has ultra‐long stability of 600 h at an industry‐level current density of 500 mA cm^−2^, significantly outperforming previously reported single‐atom catalysts. The superior performance of the synthesized samples is attributed to the synergistic regulation of lattice oxygen by oxygen vacancies and single atoms. Density functional theory (DFT) calculations reveal a novel synergistic activation mechanism that the introduction of Mo single atoms enhances the interaction between lattice oxygen and reaction intermediates. Meanwhile, the incorporation of oxygen vacancies increases the electron occupancy in anti‐bonding orbitals, further promoting lattice oxygen activation. This study establishes a direct correlation between defect engineering and single‐atom modification within the LOM pathway, providing new insights for future mechanistic investigations and practical applications.

## 2 Results and Discussion

### 2.1 Synthesis and Characterization

**Figure 1:**
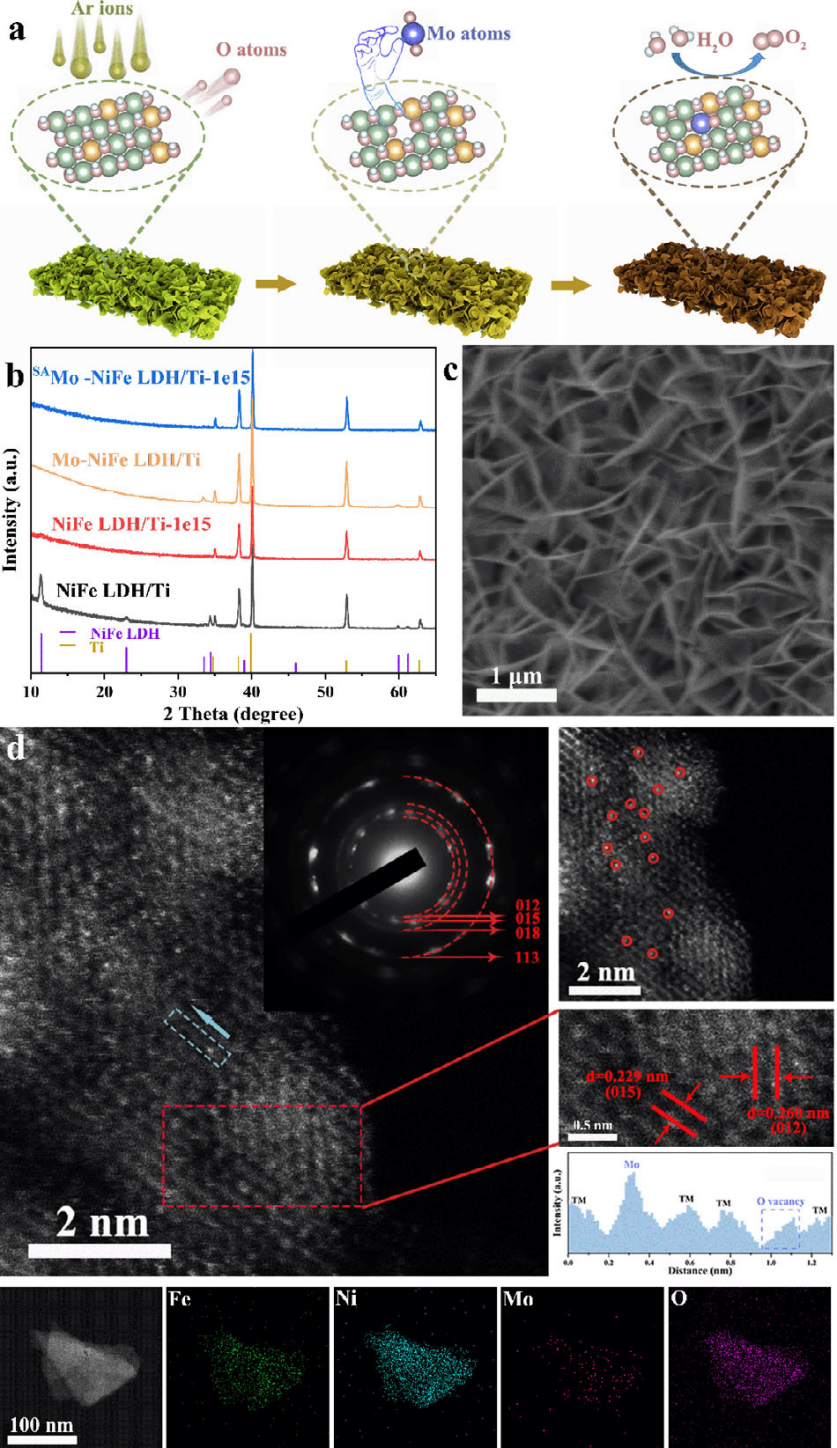
Characterization of ^SA^Mo‐NiFe LDH/Ti‐1e15. a) synthesis illustration, b) XRD patterns, c) SEM image, and d) aberration‐corrected high resolution HAADF‐STEM image of as‐synthesized sample (isolated Mo atoms are marked with red circles) with corresponding SAED pattern and EDS information.

The pristine NiFe LDH nanosheets supported on an inert substrate, titanium foil, (denoted as NiFe LDH/Ti) were synthesized by a one‐step hydrothermal method. Subsequently, high‐energy Ar^+^ ions irradiation process with the ion energy of 100 keV and the fluences of 1 × 10^14^, 5 × 10^14^, 1 × 10^15^, and 2 × 10^15^ ions cm^−2^ was carried out to fabricate defect‐rich NiFe LDH (denoted as NiFe LDH/Ti‐(fluence)). The reason for selecting Ar^+^ irradiation is to introduce appropriate defects into the catalyst while avoiding the introduction of impurity atoms that may interfere with catalytic performance. To confirm this selection, we compared the effects of commonly used irradiation ions (He^+^ and Ar^+^) on both the catalyst's structure and catalytic performance (Figures S1–S4, Supporting Information). Through these comparative analyses, Ar⁺ ions irradiation ultimately proved to be the most suitable choice. Then, the molybdenum single atoms were stabilized on the vacancy‐rich NiFe LDH/Ti‐(fluence) by the hydrothermal method (denoted as ^SA^Mo‐NiFe LDH/Ti‐(fluence)) (**Figure** **1**a). After loading Mo single atoms onto NiFe LDH samples irradiated at different fluences, we observed that the sample with fluence of 1×10^15^ ions cm^−2^ exhibits appropriate vacancy density and achieves the optimal catalytic performance (Figures S5 and S6, Supporting Information). Therefore, we focused our subsequent analysis on the sample of ^SA^Mo‐NiFe LDH/Ti‐1e15. Notably, to present the superiority of the irradiation strategy, the pristine NiFe LDH sample without irradiation was also performed to load Mo atoms for comparison (denoted as Mo‐NiFe LDH/Ti).

X‐ray diffraction (XRD) was carried out to verify the crystal structures (Figure 1b). A set of diffraction peaks located at 11.4°, 22.9°, 33.5°, 34.2°, 38.9°, 45.9°, 59.9°, and 61.3° are assigned to {003}, {006}, {101}, {012}, {015}, {018}, {110}, and {113} planes of NiFe LDH (PDF#40‐0215), respectively, indicating the successful preparation of NiFe LDH with hexagonal structure by hydrothermal method. Moreover, the SEM and STEM images with corresponding EDS mapping images in Figure 1c, Figures S7 and S8 (Supporting Information) further confirm the nanosheet morphology and structure. It is noteworthy that the intensity of the XRD peaks corresponding to the irradiated samples decreased or even disappeared, which is attributed to the irradiation damage effect induced a decrease of crystallinity by high‐energy Ar^+^ ions. Nevertheless, the SEM images (Figure S9, Supporting Information) display the consistent morphology of the nanosheets before and after irradiation.

To identify the formation of Mo single atoms dispersed on defective NiFe LDH (^SA^Mo‐NiFe LDH/Ti‐1e15) and its detailed structure feature, the spherical aberration‐corrected atomic resolution high‐angle annular dark‐field scanning transmission electron microscopy (HAADF‐STEM) was used. As displayed in Figure 1d, there are two groups of distinctive lattice fringes with the distances of the crystal plane of 0.229 and 0.260 nm, which correspond to the {015} and {012} planes of ^SA^Mo‐NiFe LDH/Ti‐1e15 catalyst, respectively. Moreover, the selected area electron diffraction (SAED) with four diffraction rings of {012}, {015}, {018}, and {113} planes are in line with the diffraction peaks of 34.4°, 38.9°, 45.9°, and 61.2° (PDF#40‐0215), respectively. These results suggest that the sample maintains its original crystal structure after the generation of vacancies and anchoring with single atoms, meanwhile maintaining its original morphology (Figure S10, Supporting Information). Notably, the high resolution HAADF‐STEM images clearly reveal that the Mo single atoms homogeneously disperse as bright spots on the nanosheets, and the profile line scan along the blue rectangle of Figure 1d clearly displays the atomic dispersion of Mo, as well as the existence of oxygen vacancy, indicating the coordination environment of single atom and vacancy. The corresponding element mapping also displays a uniform distribution of Ni, Fe, Mo, and O elements in ^SA^Mo‐NiFe LDH/Ti‐1e15 catalyst. Inductively coupled plasma optical emission spectrometry (ICP‐OES) was conducted to analyze the element content of the samples. As shown in Table S1 (Supporting Information), the relative contents of Ni and Fe elements remain almost unchanged after irradiation, while the content of Ni in the samples loaded with Mo single atoms decreased, thus, it can be inferred that Mo atoms mainly occupy the Ni sites. The content of Mo in ^SA^Mo‐NiFe LDH/Ti‐1e15 reaches 7.4 wt.%, which is larger than the majority of reported loadings (Table S2, Supporting Information) and that of the Mo‐NiFe LDH/Ti (2.6 wt.%). The above analysis indicates that the irradiation‐introduced abundant vacancies anchored Mo single atoms on NiFe LDH more easily, while partial oxygen vacancies of unanchored Mo atoms play a role in limiting the migration and agglomeration of Mo single atoms, which is beneficial for the good stability of our catalyst. Moreover, the close existence of Mo single atoms and vacancies may trigger their synergistic effect and affect catalytic activity.

**Figure 2:**
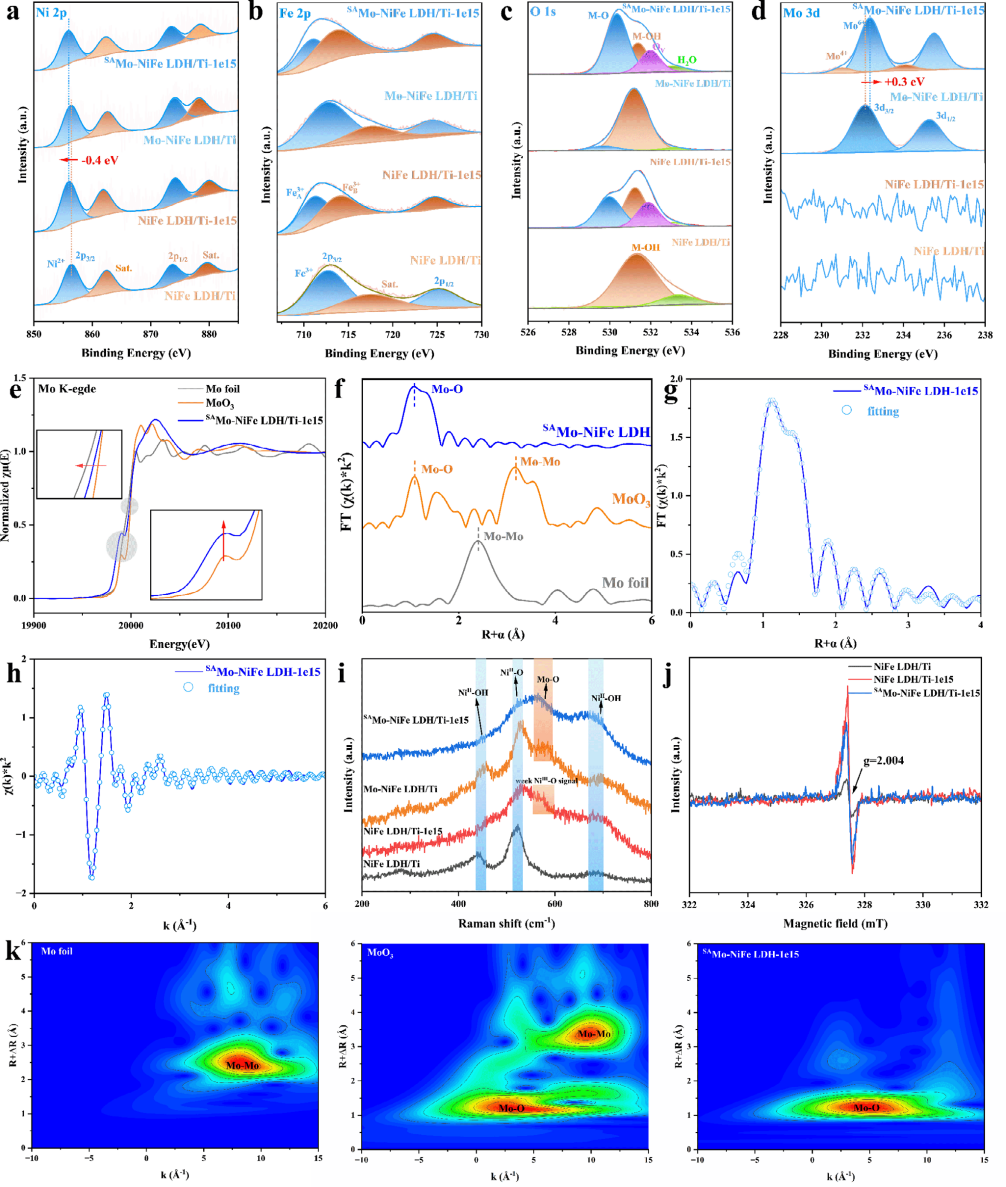
Compositional structure analysis of ^SA^Mo‐NiFe LDH/Ti‐1e15. High‐resolution XPS spectra of a) Ni 2p, b) Fe 2p, c) O 1s, and d) Mo 3d of the synthesized samples, e) XANES spectra at Mo K‐edge of ^SA^Mo‐NiFe LDH/Ti‐1e15, Mo foil, and MoO_3_, Fourier‐transform EXAFS spectra from f), g,h) Fitting curves in *R* and *k* space of the sample ^SA^Mo‐NiFe LDH/Ti‐1e15. i) Raman spectra, j) ESR results. k) Wavelet transformation of the k^2^‐weighted Mo K‐edge EXAFS signals of Mo foil, MoO_3_, and ^SA^Mo‐NiFe LDH/Ti‐1e15.

X‐ray photoelectron spectroscopy was utilized to reveal the chemical composition, oxidation states, and electronic structure of the samples. The typical peaks in the high‐resolution XPS spectra (**Figure** **2**a) located at 856.3 and 873.4 eV can be ascribed to the signals of Ni 2p_3/2_ and Ni 2p_1/2_, respectively, corresponding to the oxidation state of Ni^2+^. It is noteworthy that the peak of Ni 2p_3/2_ in the irradiated samples showed a negative shift of 0.4 eV, which is due to the influence of the generation of oxygen vacancies (O_V_). This also demonstrates that ion irradiation can alter the electronic state of Ni by introducing oxygen vacancies. According to the fine‐scan Fe 2p XPS spectra shown in Figure 2b, the peak at 712.5 eV in the pristine NiFe LDH is ascribed to the state of Fe^3+^, which is consistent with the previous reports.^[^ ^37^, ^38^, ^39^ ^]^ Notably, the peak affiliated with Fe 2p_3/2_ separates into two peaks in NiFe LDH/Ti‐1e15, which are also attributed to the Fe^3+^. This result suggests that the Fe^3+^ species exist in two different coordination environments. This phenomenon occurs due to the creation of oxygen vacancies by irradiation, which can be observed clearly in the O 1s fine spectra, confirming the emergence of oxygen vacancies after irradiation. This largely alters the electronic structure around Fe species, leading to a shift of Fe^3+^ valence states toward lower binding energy, resulting in $Fe_{A}^{3+}$. Additionally, partial bonds that connects O‐H and Fe are broken by the bombardment of high‐energy Ar^+^ ions, causing a shift of Fe^3+^ valence states toward higher binding energy, thereby resulting in $\;Fe_{B}^{3+}$. Moreover, the introduction of oxygen vacancies also leads to the decrease of satellite peaks in the Fe 2p spectrum, which further confirms the influence of oxygen vacancies on the electronic structure of Fe.^[^ ^40^, ^41^, ^42^ ^]^ Specifically, the O 1s spectrum generally exhibits four distinct binding energy states, corresponding to M‐O, M‐OH, O_V_, and adsorbed H_2_O, respectively, each of these states possesses a specific binding energy.The O 1s core‐level spectra for NiFe LDH/Ti (Figure 2c) can be divided into two spin–orbit peaks located at 531.3 and 533.4 eV, which can be attributed to the metal‐oxyhydroxide bond (M‐OH, including NiFe‐OH bonds) and oxygen in the surface‐absorbed water molecule, respectively. Then after the irradiation, part of O‐H bonds in the sample were damaged, leading to the appearance of the peaks of lattice oxygen (M‐O, including NiFe‐O bonds) as well as oxygen vacancies, which were located at 529.9 and 531.9 eV, respectively. In addition, a slight increase in the lattice oxygen peak was observed after loading Mo atoms on the unirradiated NiFe LDH, which is due to the formation of Mo‐O bonds, enhancing the intensity of M‐O signal. The ratio of the peak areas between M‐O and M‐OH indicates that Mo atoms are only loaded in small amounts for the Mo‐NiFe LDH/Ti. However, in the irradiated sample of ^SA^Mo‐NiFe LDH/Ti‐1e15, the peak intensity of the lattice oxygen is significantly enhanced, which can be attributed to a higher loading of Mo single atoms. Furthermore, the position of the lattice oxygen peak in ^SA^Mo‐NiFe LDH/Ti‐1e15 shifts to a higher binding energy compared to those of other samples. This can be attributed to the successful loading of high valence state Mo species, which affects the electronic state of oxygen in the sample. The Mo 3d fine spectra (Figure 2d) indicate that Mo species primarily exist as the oxidation state of Mo^6+^ after the loading on the pristine NiFe LDH, while in the sample of ^SA^Mo‐NiFe LDH/Ti‐1e15, there also appears a low valence state of Mo species. This occurrence of Mo^4+^ states is attributed to the presence of oxygen vacancies around the Mo single atoms. All the XPS results indicate the synergistic interaction between oxygen vacancies and Mo single atoms.

To further determine the valence state and local coordination structure of Mo species in the ^SA^Mo‐NiFe LDH/Ti‐1e15 at the atomic level, X‐ray absorption near‐edge structure (XANES) spectroscopy and Fourier‐transformed extended X‐ray absorption fine structure (EXAFS) spectra were conducted.^[^ ^43^, ^44^ ^]^ The Mo K‐edge XANES spectra of ^SA^Mo‐NiFe LDH/Ti‐1e15 in Figure 2e display the edge energy between Mo foil and MoO_3_, indicating the intermediate valence state of Mo between 0 and +6, which is consistent with the results of the XPS spectra. Notably, the increased intensity of pre‐edge peaks for Mo (Figure 2e, inset), indicated that the apportion of metal centers was in low‐coordination asymmetric configurations, providing further evidence for the existence of defect around the metal site.^[^ ^10^, ^28^ ^]^ We further performed FT‐EXAFS curve fitting in R‐space and k‐space to analyze the local atomic coordination surrounding Mo single atoms (Figure 2f–h and Table S3, Supporting Information). The optimal EXAFS fitting results indicate that Mo atom in the ^SA^Mo‐NiFe LDH/Ti‐1e15 sample is coordinated with 4.9 O atoms, confirming that the coordination of Mo atom is unsaturated. Moreover, compared to Mo foil and MoO_3_, no characteristic peaks corresponding to Mo‐Mo or Mo‐O‐Mo bonds were observed, demonstrating that Mo in ^SA^Mo‐NiFe LDH/Ti‐1e15 exists as isolated single atoms. This provides a fundamental basis for investigating the influence of single atoms on lattice oxygen. Additionally, in the Mo K‐edge EXAFS wavelet transform (WT) spectra (Figure 2k), ^SA^Mo‐NiFe LDH/Ti‐1e15 exhibits a distinct peak at 4.8 Å^−1^, indicating the presence of only a single Mo‐O coordination shell. This result is consistent with the previous analyses, further verifying that Mo single atoms are uniformly dispersed on the support.

To verify the details of the structure changes in NiFe LDH due to the introduction of vacancies by irradiation, Raman spectroscopy was performed. As shown in Figure 2i, the pristine NiFe LDH primarily exhibits three peaks at 441, 524, and 695 cm^−1^ in Raman spectrum, where the vibrations at 441 and 695 cm^−1^ correspond to Ni^II^‐OH stretching modes (Ni^II^ is consistent with Ni^2+^ species and subsequent Ni^III^ represents the Ni^3+^ species), and the peak at 524 cm^−1^ is associated with Ni^II^‐O stretching vibration. The Raman spectra demonstrate that the Ni^II^‐OH bond vibration peak at 441 cm^−1^ becomes significantly weaker, and the half‐width of peak ≈524 cm^−1^ expands after the ion irradiation, indicating a decrease in crystallinity. This confirms that ion irradiation can introduce abundant defects in NiFe LDH, which is consistent with the XRD results of the irradiated samples. Additionally, for the Mo single‐atom loaded NiFe LDH, a red‐shift in the Ni^II^‐O bond peak due to the influence of the Mo atom on the Ni^II^‐O vibrations is observed. A new peak emerging at 582 cm^−1^ can be attributed to the Mo‐O stretching vibration,^[^ ^45^ ^]^ confirming the presence of Mo‐O bond. Although a weak signal of this new Raman peak was observed in the NiFe LDH/Ti‐1e15 sample, overlapping with the Mo‐O vibrational mode, we distinguished it from the Mo‐O peak. The weak intensity of this peak in NiFe LDH/Ti‐1e15 is attributed to the formation of Ni^III^‐O species resulting from the cleavage of Ni‐OH bonds, as evidenced by the Ni 2p XPS spectra. Unlike Fe, which exhibits two distinct oxidation states, Ni shows only a shift toward lower binding energy, indicating the predominance of Ni^II^‐O vibrations. The absence of a high binding energy state corresponding to Ni^III^‐O in the Raman spectra suggests that its signal is too weak, and Ni remains primarily in the Ni^II^‐O form. Furthermore, the more pronounced presence of this peak in the Mo‐NiFe LDH/Ti sample, which lacks Ni‐OH bond cleavage, supports the conclusion that the observed Raman feature in both ^SA^Mo‐NiFe LDH/Ti‐1e15 and Mo‐NiFe LDH/Ti samples is primarily due to Mo‐O vibration. Comparing ^SA^Mo‐NiFe LDH/Ti‐1e15 with the Mo‐NiFe LDH/Ti, the peak shifts for Ni^II^‐OH are observed, which can be attributed to the effect of vacancies. Therefore, the Raman spectroscopy indicates that the presence of vacancies affects the structure configuration and enables changes in the local atomic environment.

The electron paramagnetic resonance (EPR) results provide direct evidence of the presence and evolution of oxygen vacancies in the catalyst system (Figure 2j).^[^ ^46^, ^47^, ^48^ ^]^ In the pristine NiFe LDH/Ti sample, a low concentration of oxygen vacancies is detected, whereas ion irradiation significantly increases the number of these vacancies, which is consistent with XPS results. This confirms the advantage of ion irradiation technology that it can effectively induce a high and controlled concentration of oxygen vacancies. Notably, no peaks corresponding to metal vacancies were detected in the spectra.^[^ ^48^ ^]^ Additionally, from the simulation results of vacancy concentrations via SRIM (Figure S11, Supporting Information), the amount of metal vacancies is extremely low. Thus, we exclude the influence of metal vacancies in our work. Upon the incorporation of Mo single atoms, a noticeable decrease in vacancy concentration is observed, suggesting the introduction of Mo‐O bonds during the loading process. The simultaneous presence of oxygen vacancies and Mo single atoms further substantiates a strong synergistic interaction between Mo atoms and oxygen vacancies, which plays a crucial role in modulating the local electronic structure and coordination environment. This finding highlights the interplay between defect engineering and single‐atom modification, providing new insights into their cooperative effects on catalytic activity.

### 2.2 Electrocatalytic OER Performance

**Figure 3:**
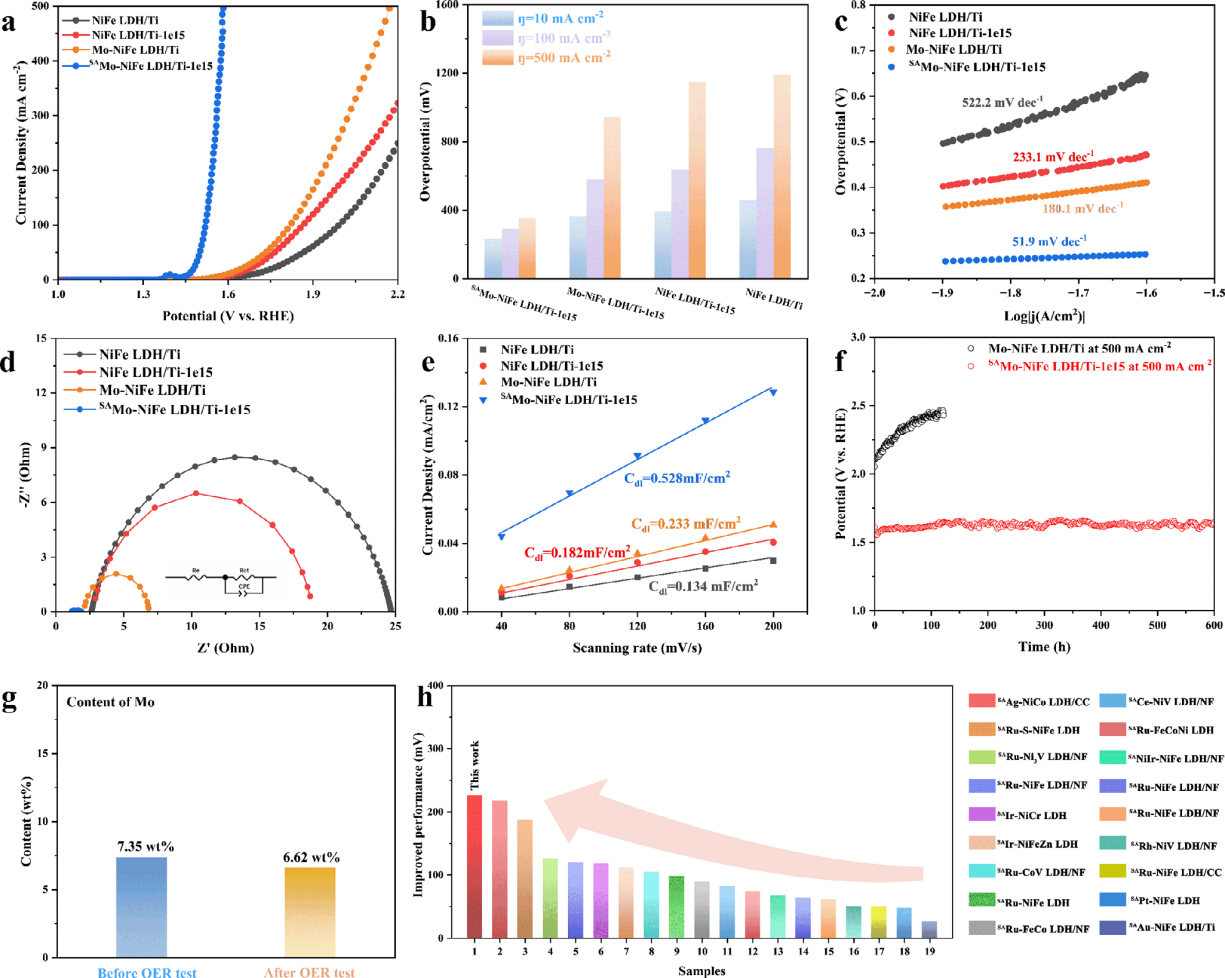
OER Performance of the synthesized samples. a) Polarization curves, b) The overpotential comparison at a current density of 10, 100, and 500 mA cm^−2^, c) Tafel plots, d) Nyquist diagrams, e) Double‐layer capacitances, f) Chronoamperometry curve, g) ICP data of metal Mo in ^SA^Mo‐NiFe LDH/Ti‐1e15 before and after OER stability test at 500 mA cm^−2^, and h) Comparison with recent studies in upgraded performance at 10 mA cm^−2^.

The OER activities of all the samples by linear sweep voltammetry (LSV) were evaluated using a standard three‐electrode system in 1 M KOH. As shown in **Figure** **3**a,b, the pristine NiFe LDH/Ti exhibits a high overpotential of 458 mV at a current density of 10 mA cm^−2^, which is consistent with the reported performance.^[^ ^37^ ^]^ However, the overpotential decreases to 393 mV at 10 mA cm^−2^ after the Ar^+^ irradiation, which is due to the introduction of vacancies under irradiation damage, activating the electrochemical reaction to a certain extent. A similar phenomenon was found in our previous work of He^+^ irradiated MoSe_2_ nanosheets.^[^ ^35^ ^]^ Strikingly, a significantly improved performance in ^SA^Mo‐NiFe LDH/Ti‐1e15 was observed, which owes to an ultra‐low overpotential of 232 mV, lower than those of NiFe LDH and commercial RuO_2_ (Figure S12, Supporting Information). However, the sample loaded with Mo atoms using unirradiated NiFe LDH has a larger overpotential of 364 mV, which is inferior in terms of Mo atoms loading and performance. This performance discrepancy in the four samples suggests that the strong interaction between vacancies and Mo atoms in ^SA^Mo‐NiFe LDH/Ti‐1e15. The catalytic performance was also optimized by regulating the irradiation fluence to control the concentration of vacancies (Figure S6, Supporting Information), all the irradiation‐assisting synthesized ^SA^Mo‐NiFe LDH/Ti exhibit better catalytic activity than those of other samples. The results above demonstrate that the ion irradiation strategy can play a positive role in the efficiency of single atomic samples. Additionally, in order to eliminate the contribution of substrate to the OER performance, the linear sweep voltammetry data of Ni foam and Ni foil were evaluated (Figure S13, Supporting Information), and the corresponding overpotentials at 10 mA cm^−2^ were 332 and 339 mV, respectively. While there is almost no catalytic activity for Ti foil, which suggests that the performance of ^SA^Mo‐NiFe LDH/Ti‐1e15, as a non‐noble metal single‐atom catalyst, still has an advantage over the reported electrocatalytic materials using Ni foam as a substrate (Table S2, Supporting Information). To evaluate the catalytic kinetics of the electrochemical reaction, Tafel plots were calculated (Figure 3c), which exhibit similar trends. In particular, ^SA^Mo‐NiFe LDH/Ti‐1e15 possesses the smallest Tafel slope of 51.9 mV dec^−1^, which indicates that the Mo single‐atom catalyst loaded by the ion irradiation strategy exhibits excellent reaction kinetics. Furthermore, electrochemical impedance spectroscopy (EIS) was carried out to estimate the charge transfer resistance (R_ct_). As displayed in Figure 3d and Table S4 (Supporting Information), NiFe LDH/Ti has a very high impedance, which is relevant to the few active sites involved in charge transfer on the surface of NiFe‐based materials with a hydrotalcite structure, causing low charge transfer efficiency. Whereas the impedance values of samples subjected to ion irradiation (NiFe LDH/Ti‐1e15) and those loaded with heteroatoms (Mo‐NiFe LDH/Ti) are reduced, which is a positive trend caused by enrichment of the active centers with vacancies and heterogeneous metal atoms, respectively. Notably, ^SA^Mo‐NiFe LDH/Ti‐1e15 anchored with single atoms employing the ion irradiation strategy possesses an extremely low impedance. This result indicates that the synergistic effect between vacancies and single atoms can enhance the efficiency of charge transfer. The electrochemical double‐layer capacitance (C_dl_) measurement is widely recognized to evaluate the electrochemically active surface area of catalysts. According to the recorded cyclic voltammetry (CV) curves at different scan rates shown in Figure S14 (Supporting Information), the calculated ESCA (Figure 3e) reveals that the pristine NiFe LDH nanosheets have the slowest process of electronic transmission with C_dl_ of 0.134 mF cm^−2^. After ion irradiation, the introduction of vacancies induces the number of active sites, thus increasing C_dl_ to 0.182 mF cm^−2^. The NiFe LDH with Mo single atoms on the surface also increases C_dl_ to 0.233 mF cm^−2^. However, ^SA^Mo‐NiFe LDH/Ti‐1e15 possesses a remarkable increased C_dl_ of 0.528 mF cm^−2^, which is 2.3 and 2.9 times higher than those of Mo‐NiFe LDH/Ti and NiFe LDH/Ti‐1e15, respectively. All these results above indicate that the synergistic effect between vacancies and single atoms largely enhances the performance of the catalyst. Notably, even with comparable Mo content to Mo‐NiFe LDH/Ti (Table S5, Supporting Information), ^SA^Mo‐NiFe LDH/Ti‐1e14 still exhibits substantial room for performance improvement, further highlighting the synergistic effect between oxygen vacancy and Mo single‐atom site.

The stability test is another important criterion for evaluating catalyst performance. We further conducted a long‐term OER test for the samples of Mo‐NiFe LDH/Ti and ^SA^Mo‐NiFe LDH/Ti‐1e15 at the high current density of 500 mA cm^−2^, respectively. As shown in Figure 3f, the OER stability of Mo‐NiFe LDH/Ti without defects is very poor, which indicates that the Mo single atoms are weakly bonded with NiFe LDH nanosheets. However, the OER performance only has slight deterioration of 30 mV after 600 h at 500 mA cm^−2^ (Figure S15, Supporting Information), which confirms that ion‐irradiation‐induced surface vacancies can firmly anchor metal single atoms and limit their loss (Figure 3g). On the other hand, DFT calculated formation energy offers crucial insights into the thermodynamic stability of the synthesized catalyst under electrochemical conditions. More negative formation energy in the sample of ^SA^Mo‐NiFe LDH/Ti‐1e15 was obtained (Figure S16, Supporting Information), indicating intrinsic robustness. We also perform ICP‐OES to confirm the stability of ^SA^Mo‐NiFe LDH/Ti‐1e15, and the Mo is only slightly dissolved. The HAADF‐STEM and corresponding EDS mapping images shown in Figure S17 (Supporting Information) confirm the preservation of Mo single atoms after the OER stability test. Finally, we compared the improved performance of ^SA^Mo‐NiFe LDH/Ti‐1e15 at a current density of 10 mA cm^−2^ with recently reported single‐atom LDH electrocatalysts. As displayed in Figure 3h and Table S2 (Supporting Information), the performance of our non‐noble metal single‐atomic electrocatalyst is comparable to the reported performance of noble metal catalysts. Moreover, the reduced overpotential of ^SA^Mo‐NiFe LDH/Ti‐1e15 compared to pristine NiFe LDH/Ti is the most noticeable.

### 2.3 Elucidation of the OER Mechanism

**Figure 4:**
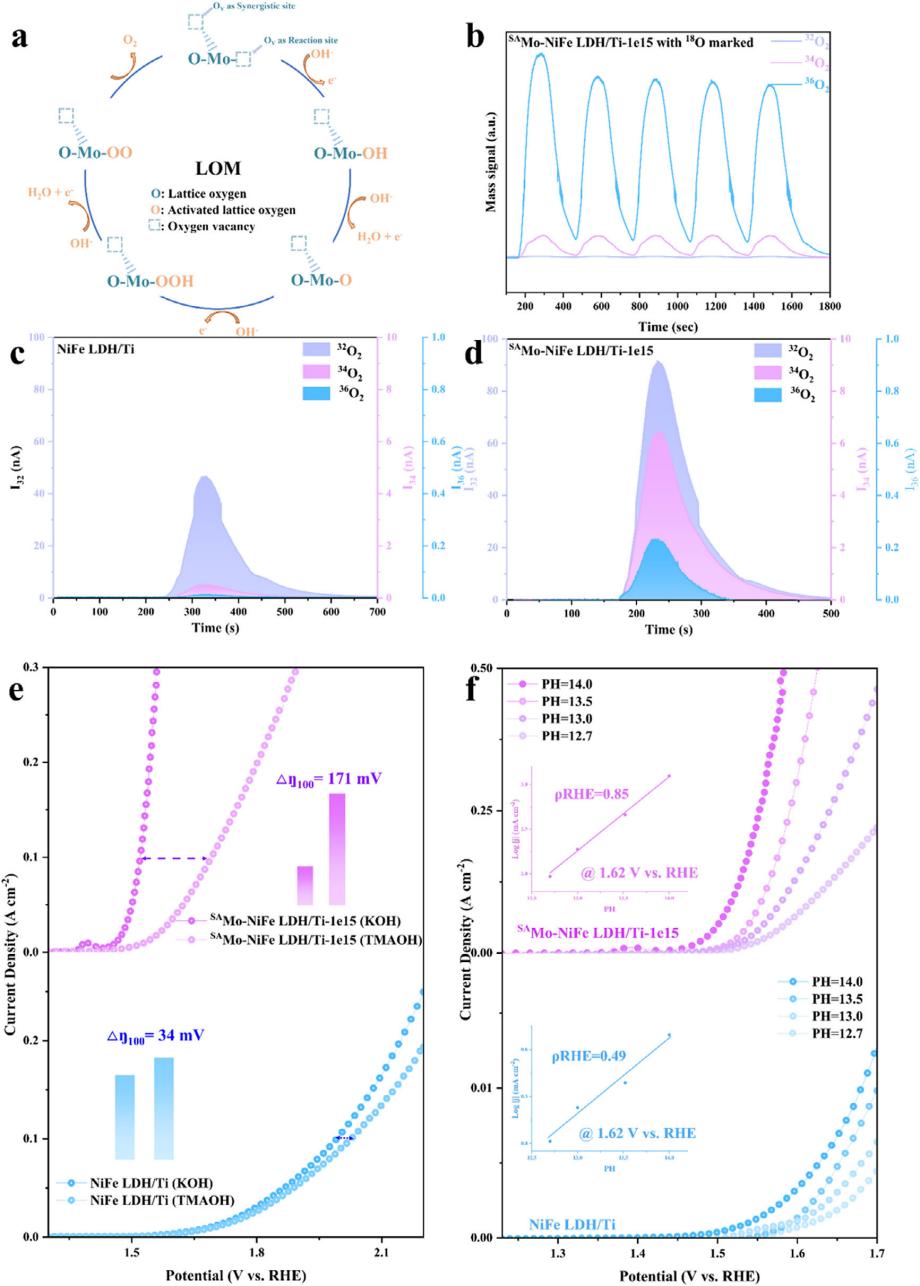
OER mechanism revealed by ^18^O labeling experiments. a) Schematic illustration of the LOM pathway on ^SA^Mo‐NiFe LDH/Ti‐1e15. b) DEMS signals of ^36^O_2_, ^34^O_2_, and ^32^O_2_ for ^SA^Mo‐NiFe LDH/Ti‐1e15 in H_2_^18^O aqueous KOH electrolyte within five times of LSV at 1.1–1.9 V (versus RHE). The differential electrochemical mass spectrometry (DEMS) signals of ^16^O_2_ (I_32_), ^16^O^18^O (I_34_), and ^18^O_2_ (I_36_) for c) NiFe LDH/Ti and d) ^SA^Mo‐NiFe LDH/Ti‐1e15. e) OER Polarization curves of NiFe LDH/Ti and ^SA^Mo‐NiFe LDH/Ti‐1e15 in 1 M KOH and 1 M TMAOH, respectively. f) OER current density at 1.62 V versus RHE for samples of ^SA^Mo‐NiFe LDH/Ti‐1e15 and NiFe LDH/Ti plotted in log scale as a function of pH, from which the proton reaction orders (ρ^RHE^ = ∂log(j)/∂pH) were calculated.

To explore the intrinsic mechanism behind the superior OER activity of Mo single atom‐loaded NiFe LDH prepared with ion irradiation assistance, we conducted a thorough review of the related studies on NiFe LDH to distinguish our mechanism from the previous research, thereby providing a better explanation for the exceptional performance and stability of our catalyst. As mentioned above, most studies on NiFe (oxy) hydroxides reported to date have focused on the AEM reaction mechanism, where metal sites serve as the active centers for OER. However, the theoretical overpotential of the OER reaction governed by the AEM mechanism is 370 mV, which is considerably higher than that of currently reported OER performance.^[^ ^12^, ^30^, ^49^, ^50^ ^]^ This inconsistency suggests that the AEM mechanism may not fully account for the high catalytic activity of NiFe‐based catalysts. Therefore, a novel LOM mechanism has been redefined to overcome the intrinsic overpotential limitations of the AEM pathway.^[^ ^29^, ^51^, ^52^, ^53^ ^]^ In the LOM pathway, the activated lattice oxygen acts as the redox center, directly participating in oxygen generation (**Figure** **4**a). By breaking the conventional scaling relationships that constrain the AEM mechanism, LOM offers a more thermodynamically favorable pathway for OER. However, most of the current research on the LOM mechanism has been focused primarily on the activation of lattice oxygen, overlooking the impact of the local coordination environment of lattice oxygen on the reaction barrier along the pathway. Therefore, we address this limitation by employing the controllable ion beam technology to manipulate local defects in the catalyst, introducing single atom sites and further regulating the coordination environment of lattice oxygen. As illustrated in Figure 4a, Figures S18 and S19 (Supporting Information), oxygen vacancies (O_V_ as reaction site) adjacent to a Mo single atom actively participate in the lattice oxygen‐mediated mechanism (LOM). In this process, OH^−^ initially adsorbs at O_V_ site, forming Mo‐OH species. Subsequent deprotonation leads to the generation of *O intermediate, which further interacts with additional OH^−^ to form *OOH species. The final deprotonation step results in the release of O_2_. In contrast, remote oxygen vacancies (O_V_ as a synergistic site), which are not influenced by Mo single atoms, remain inactive but may play a role in synergistically regulating lattice oxygen reactivity, thus achieving a synergistic coupling effect between single atoms and oxygen vacancies. ^1^⁸O‐labeling experiment was conducted to verify the involvement of lattice oxygen in the OER process of our synthesized samples. The in‐situ differential electrochemical mass spectrometry (DEMS) results, as shown in Figure 4b–d, reveal that the OER products of the ^1^⁸O‐labeled ^SA^Mo‐NiFe LDH/Ti‐1e15 in an electrolyte containing only ^1^⁶O exhibit signals corresponding to ^3^⁴O_2_ and ^3^⁶O_2_, indicating that the detected ^1^⁸O originates from the lattice oxygen within the catalyst. This finding confirms that the reaction pathway of ^SA^Mo‐NiFe LDH/Ti‐1e15 follows the LOM. Notably, NiFe LDH/Ti only displays a weak ^1^⁸O signal, while ^SA^Mo‐NiFe LDH/Ti‐1e15 exhibits a significantly larger ^1^⁶O^1^⁸O peak area and an increased ^1^⁶O^1^⁸O/^1^⁶O^1^⁶O peak area ratio (Figure S20, Supporting Information). Furthermore, the sample of ^SA^Mo‐NiFe LDH/Ti‐1e15 exhibited pronounced performance degradation compared to NiFe LDH/Ti in the tetraalkylammonium cation (TMA^+^) containing electrolyte (Figure 4e), where the TMA^+^ can suppress the LOM pathway.^[^ ^54^, ^55^ ^]^ In addition, the catalytic activity of the catalyst under the LOM pathway exhibits a stronger dependence on pH values than that under the AEM.^[^ ^12^, ^52^, ^56^ ^]^ We also conducted the LSV measurement under different pH values (PH = 12.71,13, 13.52, and 14). As illustrated in Figure 4f, it is found that the OER activity of ^SA^Mo‐NiFe LDH/Ti‐1e15 enhances significantly with increasing pH values, while NiFe LDH/Ti exhibits slight pH‐dependent activity. To precisely clarify the correlation between the activity and pH values, the proton reaction orders on RHE scale (ρ^RHE^, ρ^RHE^  =  ∂log(j)/∂pH) was used, which reflects the dependence of OER reaction kinetics on proton activity. The higher ρ^RHE^ for ^SA^Mo‐NiFe LDH/Ti‐1e15 implied a stronger pH‐dependent OER activity, which is due to the involvement of LOM mechanism activated by vacanies and Mo atoms. All these results indicate that oxygen vacancies and single atoms synergistically enhance the higher lattice oxygen activity.

**Figure 5:**
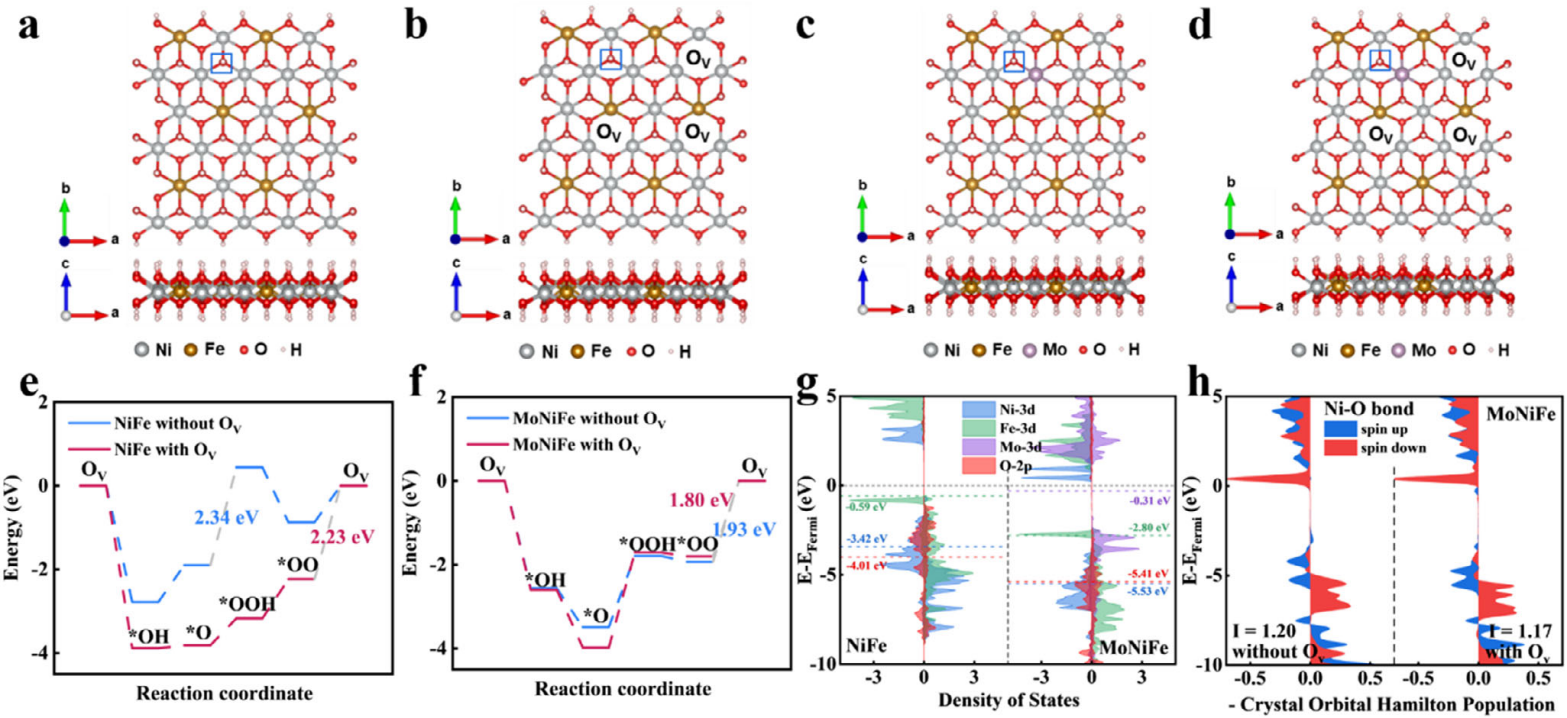
OER mechanism revealed by DFT calculations. a–d) The top and side view of NiFe LDH and MoNiFe LDH without and with oxygen vacancies. The squares represent lattice oxygen active sites. e), f) Gibbs free energy diagrams of the LOM pathway on NiFe LDH and MoNiFe LDH. g) The PDOS of NiFe LDH and MoNiFe LDH. h) Crystal orbital Hamilton populations (COHP) of the Ni‐O bond in MoNiFe LDH without and with oxygen vacancies.

To further explore the synergistic coupling role of Mo single atoms and oxygen vacancies in activating lattice oxygen, DFT calculations were performed to elucidate the synergistic interaction between single atoms and vacancies under the LOM pathway. The atomic configurations of NiFe LDH, both in the absence and presence of oxygen vacancies (O_V_), are illustrated in **Figure** **5**a,b, respectively, where the squares indicate active lattice oxygen sites. In NiFe LDH, the active sites are coordinated by three Ni atoms. To investigate the catalytic role of Mo single atoms, the active sites surrounded Ni atoms are substituted with Mo, leading to the formation of a Mo‐O bond in place of a Ni‐O bond. This is supported by ICP‐OES analysis (Table S1, Supporting Information), which shows that the relative concentration of Ni decreases with increasing Mo loading, whereas the Fe content remains unchanged. This observation strongly suggests that Mo preferentially substitutes Ni sites within the structure. The atomic structures of MoNiFe LDH without and with oxygen vacancies, are depicted in Figure 5c,d, respectively.

As shown in Figure 5e,f, in the LOM pathway, the formation of *OOH serves as the rate‐determining step (RDS) for pristine NiFe LDH, with an overpotential of 2.34 eV. The introduction of oxygen vacancies (O_V_) enhances *OOH adsorption, shifting the RDS to the O_V_ formation step with a reduced overpotential of 2.23 eV. The overpotential obtained for pristine NiFe LDH is comparable to previously reported theoretical values,^[^ ^18^, ^57^ ^]^ validating the reliability of our computational model. However, studies on NiFe LDH with O_V_ remain limited, and the associated overpotential strongly depends on both the structural configuration and the precise positions of the vacancies. To address this, we systematically explore possible O_V_ locations in this work. The final configurations are provided in the Supplementary Material, and detailed descriptions of the structural models are presented in the Methods section.

A remarkable decrease in overpotential is observed after the incorporation of Mo single atoms. In this case a synergistic effect is found, reducing the energy barrier for *OOH formation and shifting the RDS to the O_V_ formation step, thereby reducing the overpotential to 1.93 eV. Notably, the simultaneous incorporation of both Mo atoms and oxygen vacancies into MoNiFe LDH results in an additional decrease in overpotential to 1.80 eV. These findings indicate that the synergistic interaction of Mo and oxygen vacancies significantly boost the catalytic performance.

To gain deeper insight into the role of the simultaneous introduction of Mo single atoms and oxygen vacancies in NiFe LDH to enhance OER performance, the projected density of states (PDOS) of NiFe LDH and MoNiFe LDH is analyzed and presented in Figure 5g. The PDOS data was extracted from the single Ni, Fe, Mn, and O atoms located near the OER active site. In both NiFe LDH and MoNiFe LDH, the Ni‐O bond exhibits strong orbital coupling between the metal (Ni) and the oxygen atoms. However, in MoNiFe LDH, the Mo‐O bond shows much weaker orbital coupling compared to Ni‐O. This difference in orbital coupling suggests that Mo‐O bonds are weaker than Ni‐O bonds. As a result, when Mo‐O bonds partially replace Ni‐O bonds, the oxygen atoms become more labile and more prone to bond cleavage during the reaction. This effect reduces the barrier for oxygen vacancy activation, thereby facilitating lattice oxygen participation in the OER process. In addition to the electronic modulation induced by Mo incorporation revealed by PDOS, the bond strength analysis based on crystal orbital Hamilton populations (COHP) further demonstrates the effect of oxygen vacancies on Ni‐O interactions. Figure 5h displays the COHP analysis, where negative and positive values of ‐COHP correspond to anti‐bonding and bonding states, respectively. To quantitatively assess the strength of metal‐oxygen interactions, the integrated ‐COHP (I) up to the Fermi level is calculated. The Ni‐O bond integral values are 1.20 and 1.17 for MoNiFe‐LDH without and with oxygen vacancies, respectively. The introduction of oxygen vacancies in MoNiFe‐LDH leads to a shift in electron occupancy to anti‐bonding orbitals, weakening the Ni‐O bond and promoting lattice oxygen activation. Overall, Mo incorporation facilitates the activation of lattice oxygen, while the introduction of oxygen vacancies further promotes lattice oxygen participation in the reaction, resulting in a synergistic enhancement of the overall catalytic performance.

## 3 Conclusion

This work first reports an ion irradiation strategy for synthesizing a high‐loading and stable non‐noble metal single‐atom electrocatalyst with rich oxygen vacancies for efficient oxygen evolution reactions. By utilizing ion irradiation‐induced defects in the nanolayered structure of NiFe LDH as anchoring sites, Mo single atoms were successfully immobilized onto vacancy‐rich layered double hydroxides, achieving a remarkable single‐atom loading of 7.4 wt.%. Due to the synergistic effect between single atoms and oxygen vacancies, the catalyst grown on an inert Ti substrate exhibits an OER performance of 232 mV (η = 10 mA cm^−2^) and demonstrates ultralong‐term stability (>600 h) at a high current density of 500 mA cm^−2^. The ion irradiation strategy employed in this work provides a facile and precise approach for modulating surface defects, enabling the construction of high performance catalysts with optimized defect types and concentrations, thereby addressing the long‐standing low‐loading limitation of single‐atom catalysts. Furthermore, the study of local coordination environment regulation for activated lattice oxygen offers new insights for advancing beyond conventional catalyst design, paving the way for the next‐generation electrocatalysts.

## 4 Experimental Section

### Synthesis of NiFe LDH/Ti Nanosheets

NiFe LDH nanosheets were first synthesized via a hydrothermal method. To prepare the precursor solution, 0.75 mmol of nickel nitrate hexahydrate (Ni(NO_3_)_2_·6H_2_O), 0.256 mmol of iron nitrate nonahydrate (Fe(NO_3_)_3_·9H_2_O), 8 mmol of urea, and 3.2 mmol of ammonium fluoride (NH_4_F) were dissolved in 70 mL of deionized water. After ultrasonic treatment for 5 min, the solution was transferred into a 100 mL Teflon‐lined autoclave, with a 3 × 7 cm^2^ cleaned titanium foil placed inside. The autoclave was then heated in an oven at 120 °C for 4 h under hydrothermal conditions. After the reaction, the titanium foil was removed, cleaned thoroughly with deionized water and ethanol, and air‐dried. The sample was labeled as NiFe LDH/Ti.

### Preparation of Ar^+^ Ion Irradiated NiFe LDH/Ti Nanosheets

The NiFe LDH nanosheets were irradiated with 100 keV argon ions to introduce vacancies, with the concentration being controlled by varying the irradiation influence. The original NiFe LDH samples were irradiated with argon ions at the influences of 1 × 10^14^, 5 × 10^14^, 1 × 10^15^, and 2 × 10^15^ ions cm^−^^2^, respectively. The samples were labeled as NiFe LDH/Ti‐1e14, NiFe LDH/Ti‐5e14, NiFe LDH/Ti‐1e15, NiFe LDH/Ti‐2e15, respectively.

### Preparation of ^SA^Mo‐NiFe LDH/Ti‐1e15

A specific amount of ammonium molybdate (5 mg) was added to 30 mL of octanol and stirred for 1 h. The solution was then transferred to a 50 mL Teflon‐lined autoclave, where 3 × 3 cm^2^ pieces of NiFe LDH/Ti‐1e14, NiFe LDH/Ti‐5e14, NiFe LDH/Ti‐1e15, and NiFe LDH/Ti‐2e15 were placed. The autoclave was then heated in an oven at 200 °C for 24 h under hydrothermal conditions. After the reaction, the samples were washed several times with ethanol and deionized water, and then air‐dried. The samples were labeled as ^SA^Mo‐NiFe LDH/Ti‐1e14, ^SA^Mo‐NiFe LDH/Ti‐5e14, ^SA^Mo‐NiFe LDH/Ti‐1e15, and ^SA^Mo‐NiFe LDH/Ti‐2e15, respectively. The preparation of Mo‐NiFe LDH/Ti was performed in the same method.

### Structural Characterizations

X‐ray diffraction (XRD) patterns of all the samples were collected using a SmartLab 3KW X‐ray diffractometer with Cu Kα radiation (λ = 1.5405 Å). The morphology of the samples was examined using a Field‐Emission Scanning Electron Microscope (FE‐SEM, Hitachi S‐4800, 10 kV). Aberration‐Corrected Transmission Electron Microscopy (TEM, JEM‐ARM200CF, 80 kV) and corresponding Energy‐Dispersive X‐ray Spectroscopy (EDS) provided structural and compositional information. Additionally, Confocal Raman Microscope (Raman, XploRA Plus, excitation laser wavelength of 532 nm), Inductively Coupled plasma‐Atomic Emission Spectroscopy (ICP‐OES, Agilent 5110), Electron Paramagnetic Resonance (EPR, JES‐FA200), X‐ray Photoelectron Spectroscopy (XPS, ESCALAB250Xi), and X‐ray Absorption Spectroscopy (XAS) were employed for further characterization of the samples' structure and composition.

### Electrochemical Tests

Electrochemical tests were conducted using a CHI760E electrochemical workstation. A standard three‐electrode system was employed, with the prepared samples (work area at 1 × 1 cm^2^) serving as the working electrode, a graphite rod as the counter electrode to exclude the influence of typical Pt fiol, and an Ag/AgCl electrode as the reference electrode. All sample tests were conducted in 1 M KOH electrolyte solutions, which were deaerated by bubbling with N2 for 30 min, and electrode potentials were calibrated with respect to the reversible hydrogen electrode by the equation of *E*_(RHE)_ = *E*_(Ag/AgCl)_ + 0.197 + 0.0591 pH. All data were reported with iR compensation of 80%, unless otherwise specified. Linear Sweep Voltammetry (LSV) and Cyclic Voltammetry (CV) measurements were performed at a scan rate of 5 mV s^−1^. The Tafel slope was calculated by fitting the line portion of Tafel plots (*η = blog (j) + a*), where η is the overpotential and j is the current density. Electrochemical Impedance Spectroscopy (EIS) was obtained with frequency ranging from 100 000 to 0.1 Hz. The electrochemical double‐layer Capacitance (C_dl_) measurements were determined at scan rates from 40 to 200 mV s^−1^.

### ^18^O Isotope Labeling Experiment

The pristine NiFe LDH/Ti and ^SA^Mo‐NiFe LDH/Ti‐1e15, were placed in a 1 M KOH electrolyte solution prepared with H_2_^18^O and subjected to a constant voltage of 1.5 V (versus Ag/AgCl) for ^18^O labeling. Subsequently, the catalysts labeled with ^18^O were thoroughly rinsed several times with H_2_^16^O_2_ to remove the remaining H_2_^18^O.

### DEMS Analyses

The operando Differential Electrochemical Mass Spectrometry (DEMS) system (Shanghai LingLu Instrument Corp., Ltd., China) contained a PrismaPlus quadrupole mass spectrometer from Pfeiffer Vacuum and custom‐build Swagelok cells. It is a technique that combines electrochemical measurements with mass spectrometry to detect and analyze the gaseous products generated during electrochemical reactions. The DEMS analysis was conducted with the working electrode, reference electrode, and counter electrode being an ^18^O‐labeled ^SA^Mo‐NiFe LDH/Ti‐1e15, an Ag/AgCl electrode, and a Pt foil, respectively. Cyclic voltammetry (CV) measurements were performed in a 1 M KOH electrolyte at a scan rate of 5 mV s^−1^, while the gaseous products were monitored via mass spectrometry.

### First‐Principles Calculations

First‐principles calculations were performed within the framework of density functional theory (DFT) using the Vienna Ab initio Simulation Package (VASP).^[^ ^58^, ^59^ ^]^ The Kohn‐Sham equations were solved employing the projector augmented wave (PAW) approach.^[^ ^60^ ^]^ The exchange‐correlation interactions were described using the Perdew‐Burke‐Ernzerhof (PBE) functional within the generalized gradient approximation (GGA).^[^ ^61^ ^]^ To account for the strong electron correlations in transition metals, Hubbard U corrections were applied to Ni, Fe, and Mo, with effective U values of 6.20, 5.30, and 4.38 eV, respectively, as obtained from the Materials Project database (https://materialsproject.org), which have been shown to agree well with experimental data and previous literature.^[^ ^62^ ^]^ The partial occupancies of electronic states were determined using Gaussian smearing with a width of 0.1 eV. In all DFT calculations, the surface models were constructed using a 6 × 5 × 1 supercell, containing 120 atoms in total. The slab consisted of 6 layers along the [001] crystallographic direction with a thickness of ≈15 Å, and a vacuum region of 10 Å was applied in both the [001] and [010] directions to prevent spurious interactions, as shown in Figure S18 (Supporting Information). Structural optimizations were carried out using a Monkhorst‐Pack *k*‐point mesh of 1 × 1 × 1 for Brillouin zone sampling, with a plane‐wave energy cutoff set to 500 eV. To incorporate dispersion interactions, the DFT‐D3 correction scheme was utilized. The convergence criteria were set to 10^−5^ eV for electronic self‐consistency and 0.02 eV Å^−1^ for ionic relaxation. For the partial density of states (PDOS) calculations, a 3 × 3 × 3 *k*‐point mesh was employed. The crystal orbital Hamilton population (COHP) analysis was conducted for selected atomic pairs using the Lobster software package.^[^ ^63^, ^64^ ^]^

## Conflict of Interest

The authors declare no conflict of interest.

## Supporting information



Supporting Information

## Data Availability

The data that support the findings of this study are available in the supplementary material of this article.
