# Supplementary material for: Dual‐Site Synergistic Mechanism via Single‐Atom and Vacancy Drives Lattice Oxygen Activation in Layered Double Hydroxides
Source: Adv Sci (Weinh). 2025 Dec 5;13(11):e15407. doi: 10.1002/advs.202515407 (PMC12931252; doi:10.1002/advs.202515407)
Supplement: Supplementary file 1 — Supporting Information [file ADVS-13-e15407-s001.docx]

Supporting Information

Dual-site synergistic mechanism via single-atom and vacancy drives lattice oxygen activation in layered double hydroxides

Shixin Wu^1, #^, Wenyu Lu^2, #^, Shijun Zhao^2,^*, Kai Zhao^1^, Ning Yan^1^, Liqiu Huang^1^, Derun Li^1^, Tao Jiang^1^, Hengyi Wu^1^, Feng Ren^1,3,^*

**Supplementary Text**

**Theoretical calculations**

The present investigation encompasses lattice oxygen mechanism (LOM) for oxygen evolution reaction (OER) analysis. The activation barriers for OER across various surfaces were evaluated using the computational hydrogen electrode (CHE) approach, where electrode kinetics were derived from adsorption Gibbs free energies obtained from density functional theory (DFT) calculations, with the electrode potential referenced to the normal hydrogen electrode (NHE). Thermodynamic corrections for gaseous H_2_ and liquid H_2_O were implemented at standard conditions of 298.15 K and pressures of 1.0 bar and 0.035 bar, respectively, based on vibrational frequency analysis. Due to inherent limitations in DFT regarding the treatment of molecular oxygen, the Gibbs free energy of O_2_ was determined by the following relation:

*G*_O2_ = 2*G*_H2O_ – 2*G*_H2_ + 4.92 eV (1)

The LOM mechanism involves four distinct electrochemical steps:

O_V_ + OH^–^ → *O*_l_*H + e^–^, (2)

*O*_l_*H + OH^–^ → *O*_l_* + H_2_O + e^–^, (3)

*O*_l_* + OH^–^ → *O*_l_*OH + e^–^, (4)

*O*_l_*OH + OH^–^ → *O*_l_*O + H_2_O + e^–^, (5)

*O*_l_*O → O_V_ + O_2_ (g), (6)

where * represents the vacancy sites. O*_l_* denotes the lattice oxygen atoms. The corresponding atomic configurations used in this study are presented in Supplementary Fig. 1. The thermodynamic barriers for the LOM pathway were evaluated through:

Δ*G*_1_ = *G*(*O*_l_*H) + 0.5 *G*(H_2_) – *G*(H_2_O) – *G*(O_V_) – *eU*, (7)

Δ*G*_2_ = *G*(*O*_l_*) + 0.5 *G*(H_2_) – *G*(*O*_l_*H) – *eU*, (8)

Δ*G*_3_ = *G*(*O*_l_*OH) + 0.5 *G*(H_2_) – *G*(H_2_O) – *G*(*O*_l_*) – *eU*, (9)

Δ*G*_4_ = *G*(*O*_l_*O) + 0.5 *G*(H_2_) – *G*(*O*_l_*OH) – *eU*, (10)

Δ*G*_5_ = *G*(*) + *G*(O_2_) – *G*(*O*_l_*O), (11)

where *U* is the potential with respect to the normal hydrogen electrode (NHE).

The LOM overpotential was subsequently determined by:

*η* = Max {Δ*G*_1_, Δ*G*_2_, Δ*G*_3_, Δ*G*_4_, Δ*G*_5_} (12)

To evaluate the stability of the LDH samples, the formation energies of NiFe and MoNiFe LDHs were calculated according to the following equation:

$E_{f}=E_{slab}-\sum_{i} c_{i}E_{i}$ (13)

where 𝐸_slab_ is the total energy of the NiFe or MoNiFe slab model, and *c_i_* and *E_i_* represent the concentration and the energy of the corresponding *i*th component (NiOOH, FeOOH, or MoOOH), respectively.


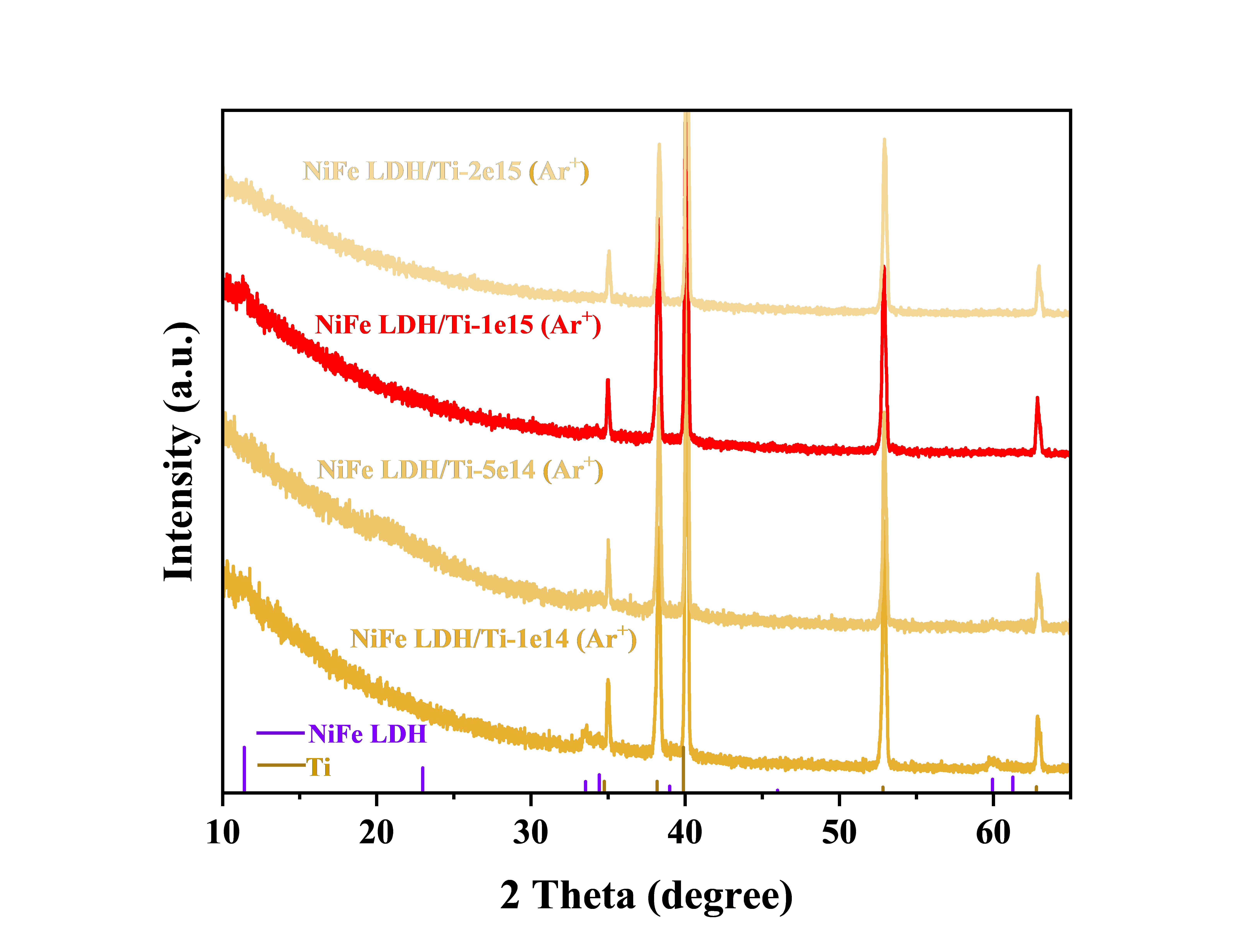


**Figure S1.** XRD patterns for NiFe LDH samples irradiated at fluences of 1×10^14^, 5×10^14^, 1×10^15^, and 2×10^15^ ions cm^-2^.


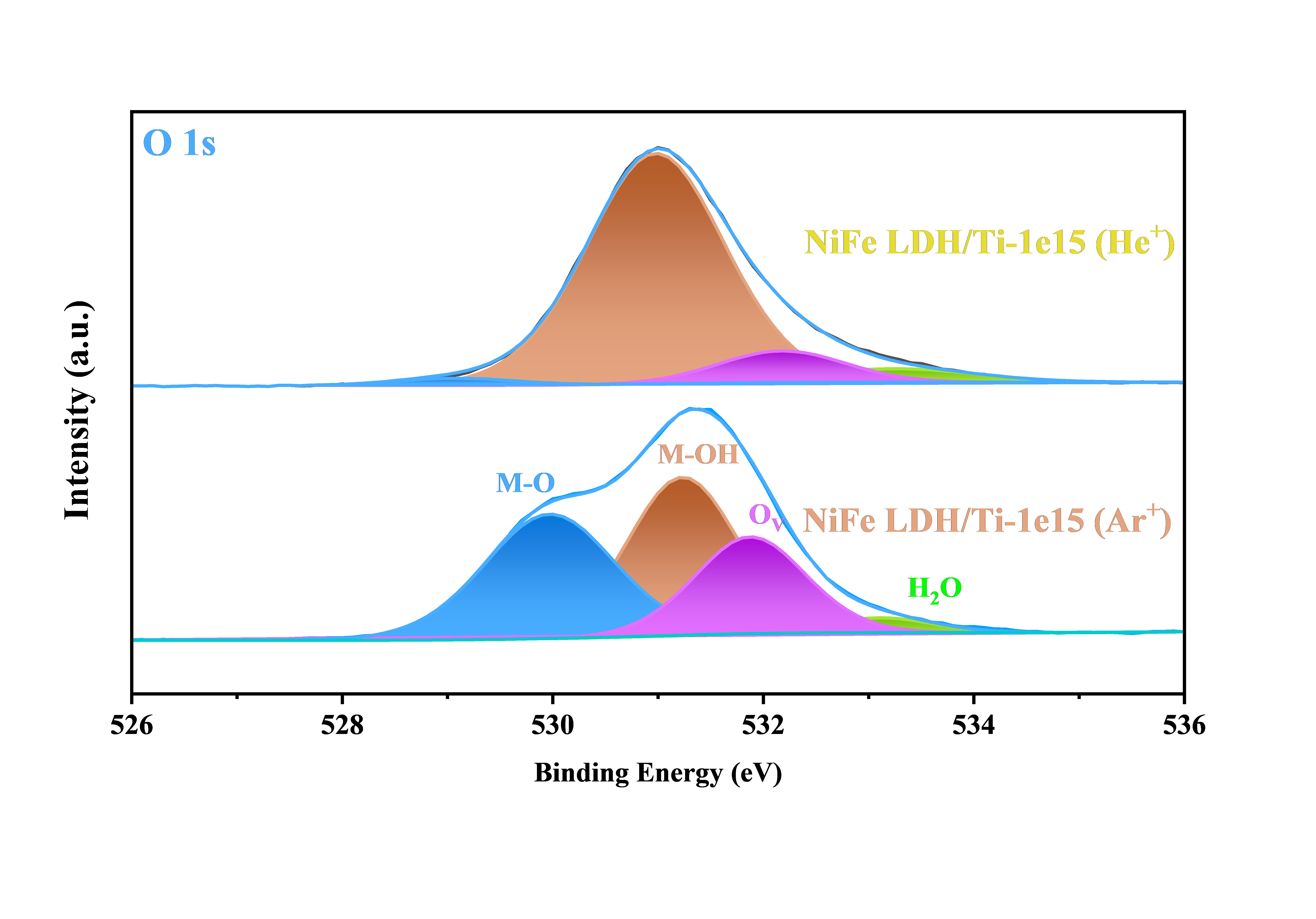


**Figure S2.** High-resolution XPS spectra of O 1s for the samples of NiFe LDH irradiated with He^+^ and Ar^+^ ions, respectively.
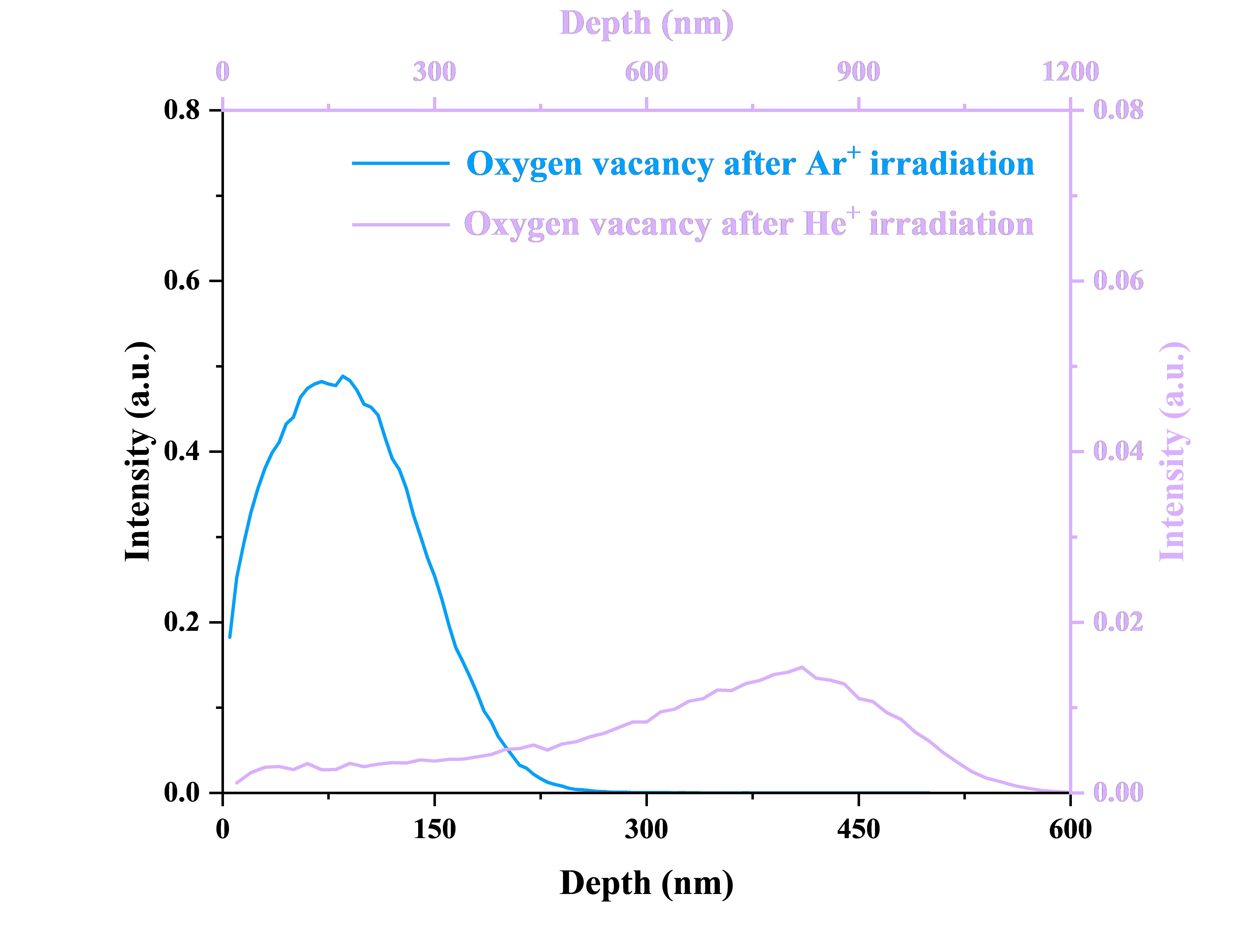


**Figure S3.** The oxygen vacancy concentration distribution simulated via SRIM for Ar^+^ and He^+^ ions irradiated samples.


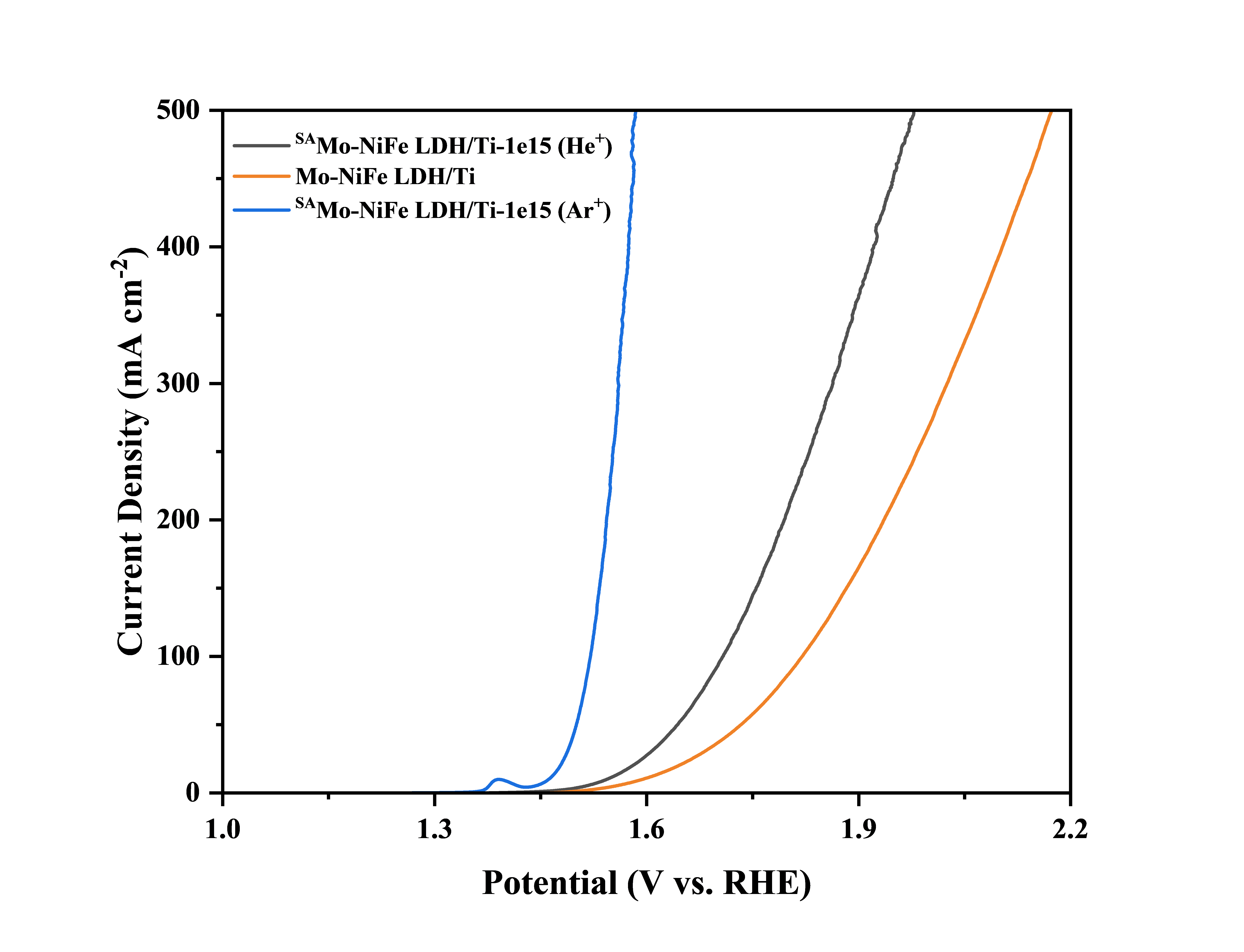


**Figure S4.** OER Polarization curves of ^SA^Mo-NiFe LDH/Ti-1e15 (He^+^), Mo-NiFe LDH/Ti, and ^SA^Mo-NiFe LDH/Ti-1e15 (Ar^+^) for comparison.


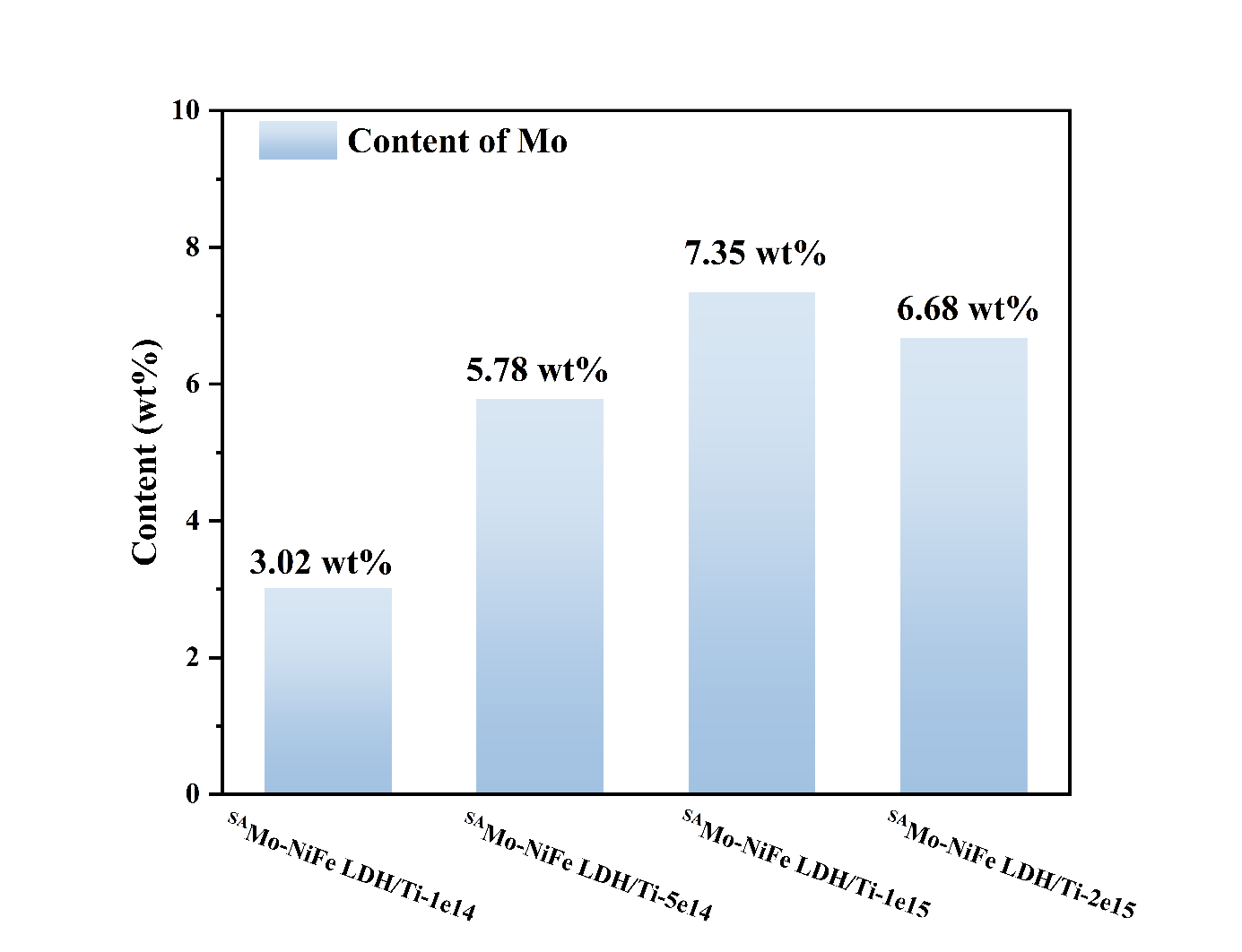


**Figure S5.** ICP data of metal Mo in ^SA^Mo-NiFe LDH/Ti-(1e14, 5e14, 1e15, 2e15).


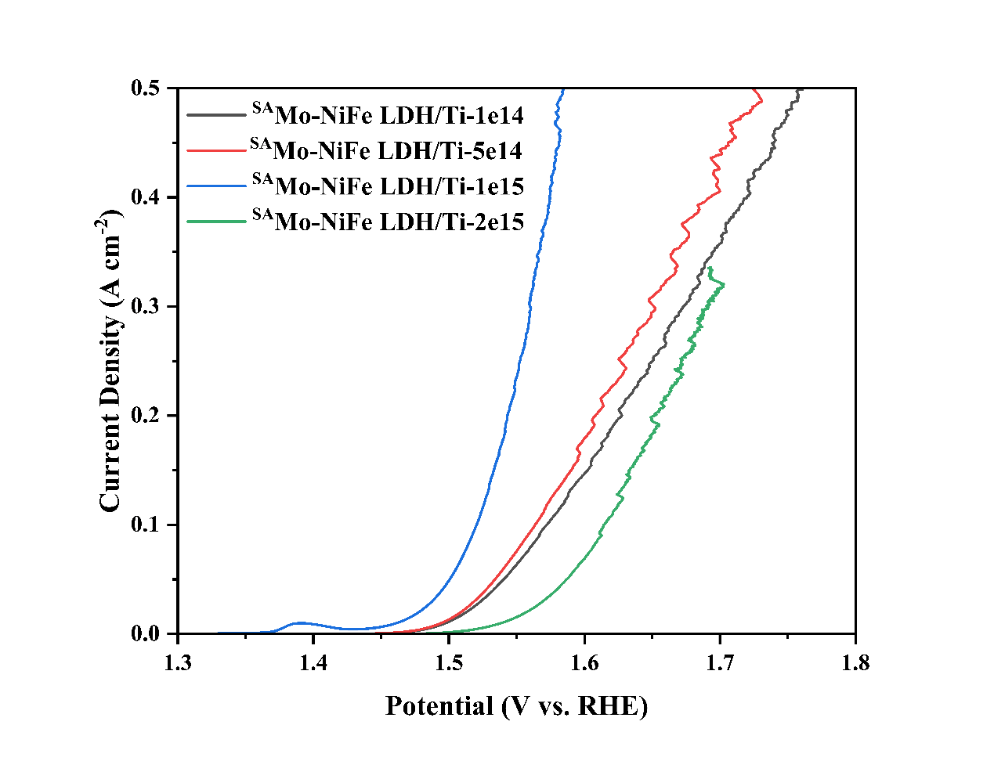


**Figure S6.** OER Polarization curves of ^SA^Mo-NiFe LDH/Ti-1e14, ^SA^Mo-NiFe LDH/Ti-5e14, ^SA^Mo-NiFe LDH/Ti-1e15, ^SA^Mo-NiFe LDH/Ti-2e15 for comparison.


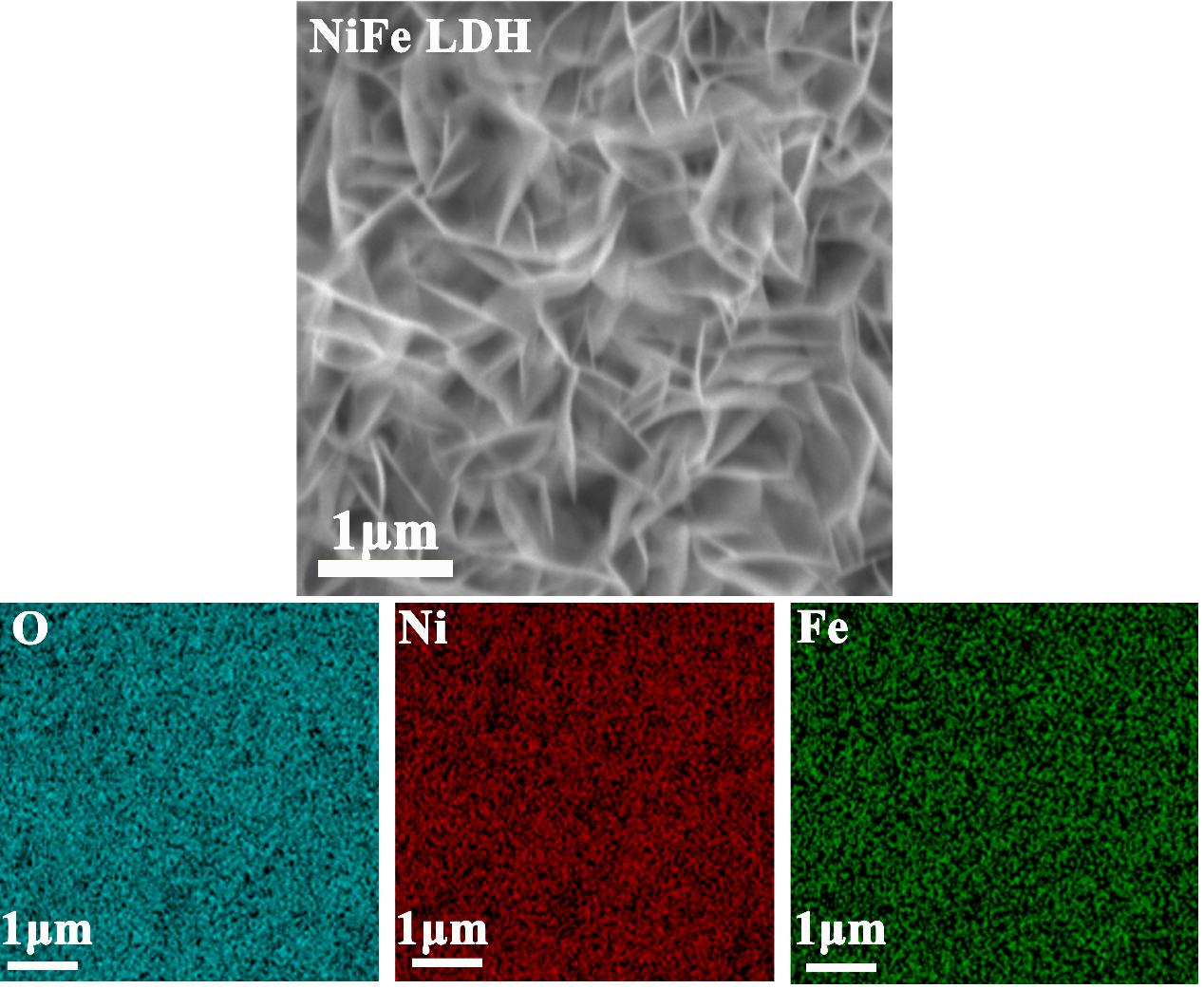


**Figure S7.** SEM image and corresponding EDS mapping of NiFe LDH/Ti .


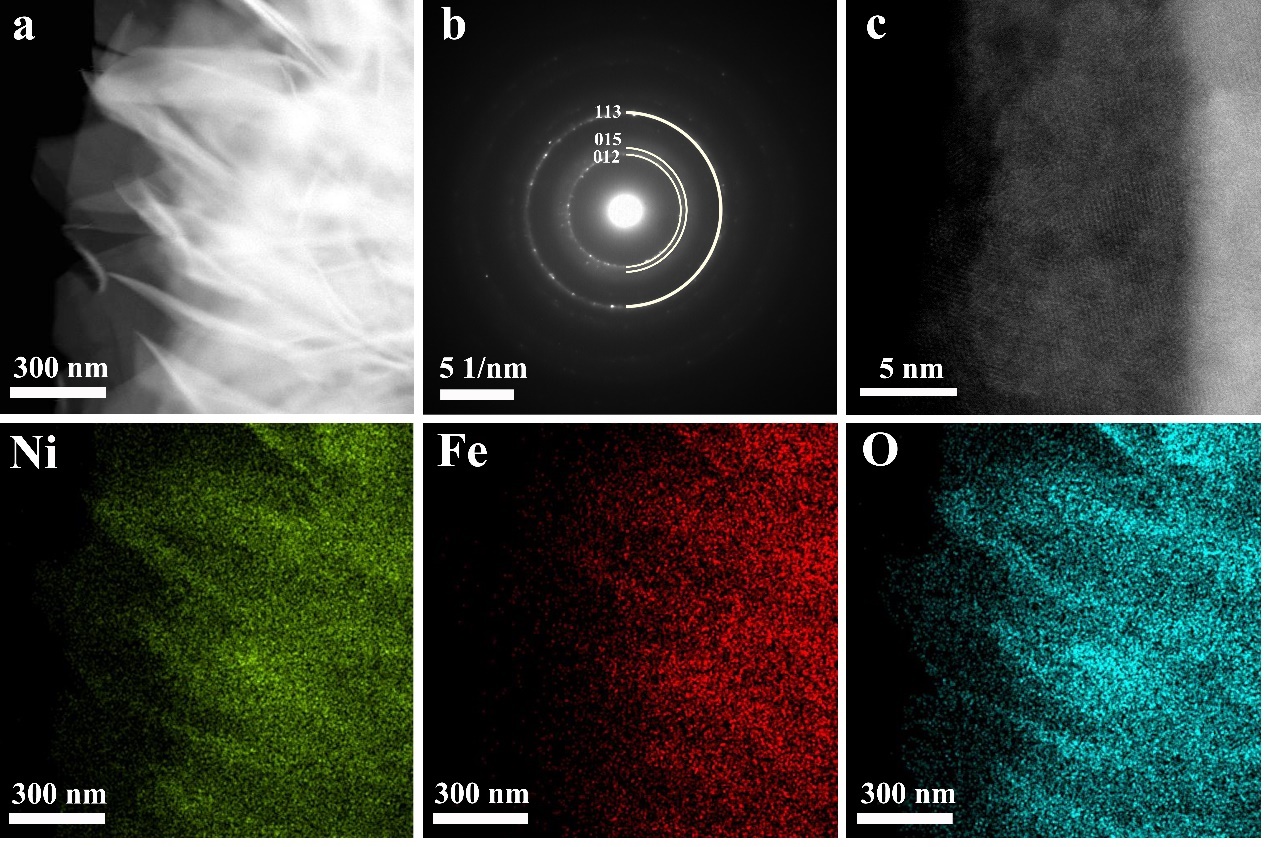


**Figure S8.** a) HAADF-STEM image of NiFe LDH/Ti, b) SAED pattern, c) high-resolution HAADF-STEM image, and corresponding EDS mapping images of a).


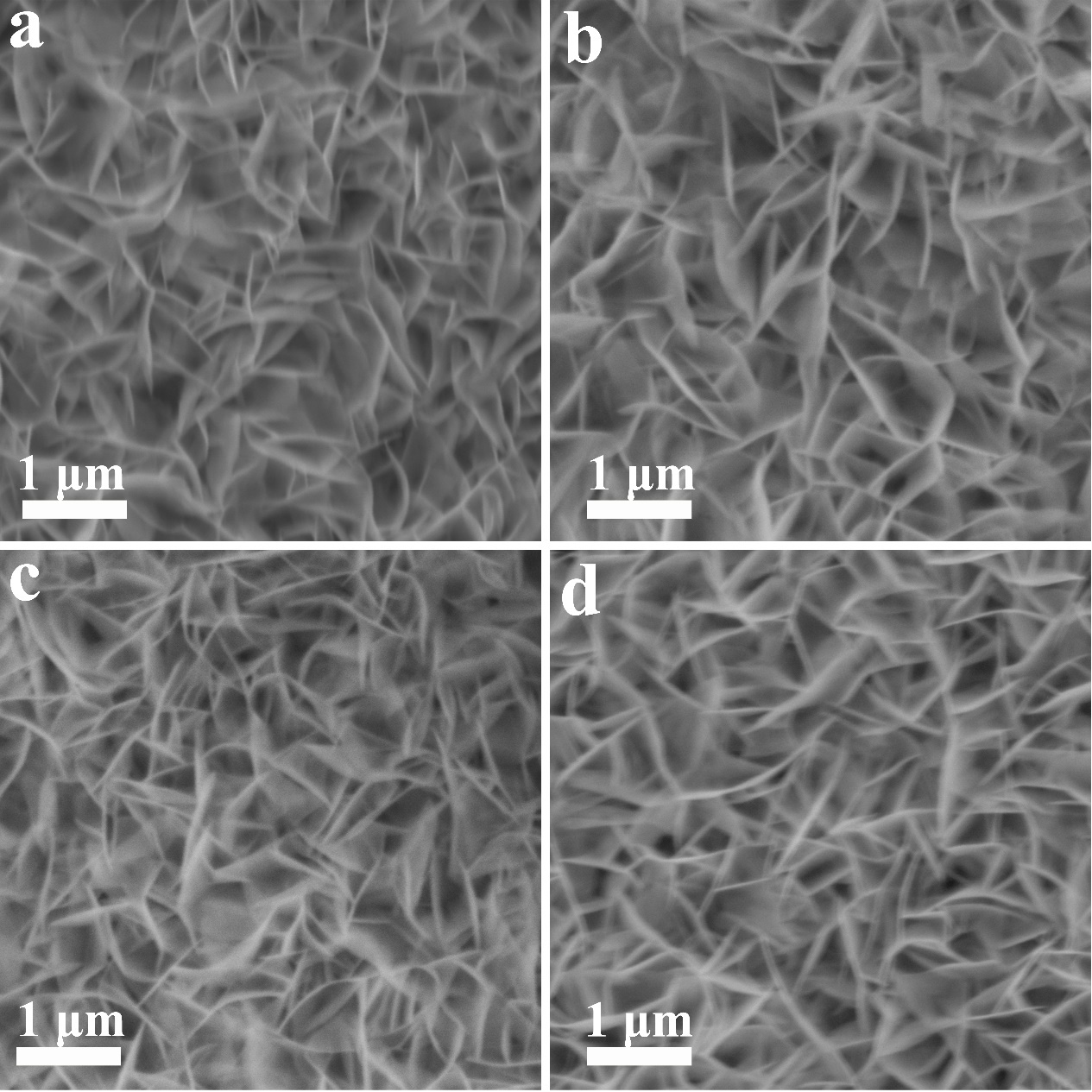


**Figure S9.** SEM images of a) NiFe LDH/Ti, b) NiFe LDH/Ti-1e15, c) Mo-NiFe LDH/Ti, and d) ^SA^Mo-NiFe LDH/Ti-1e15.
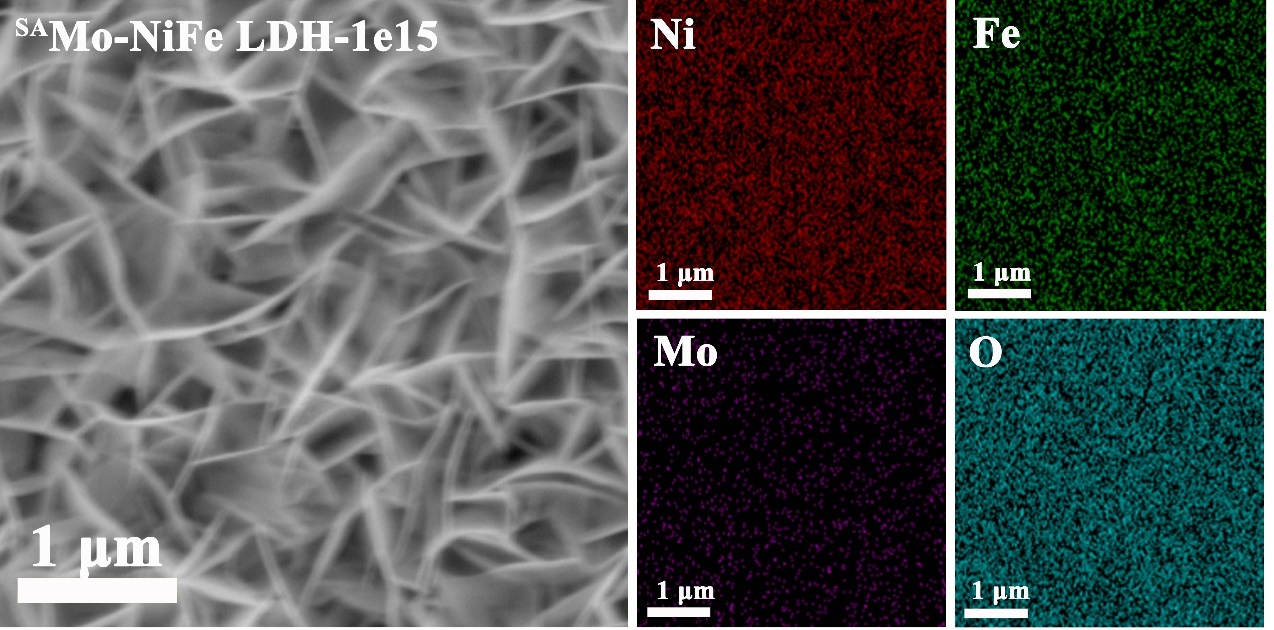


**Figure S10.** SEM and corresponding EDS mapping images of ^SA^Mo-NiFe LDH/Ti-1e15.


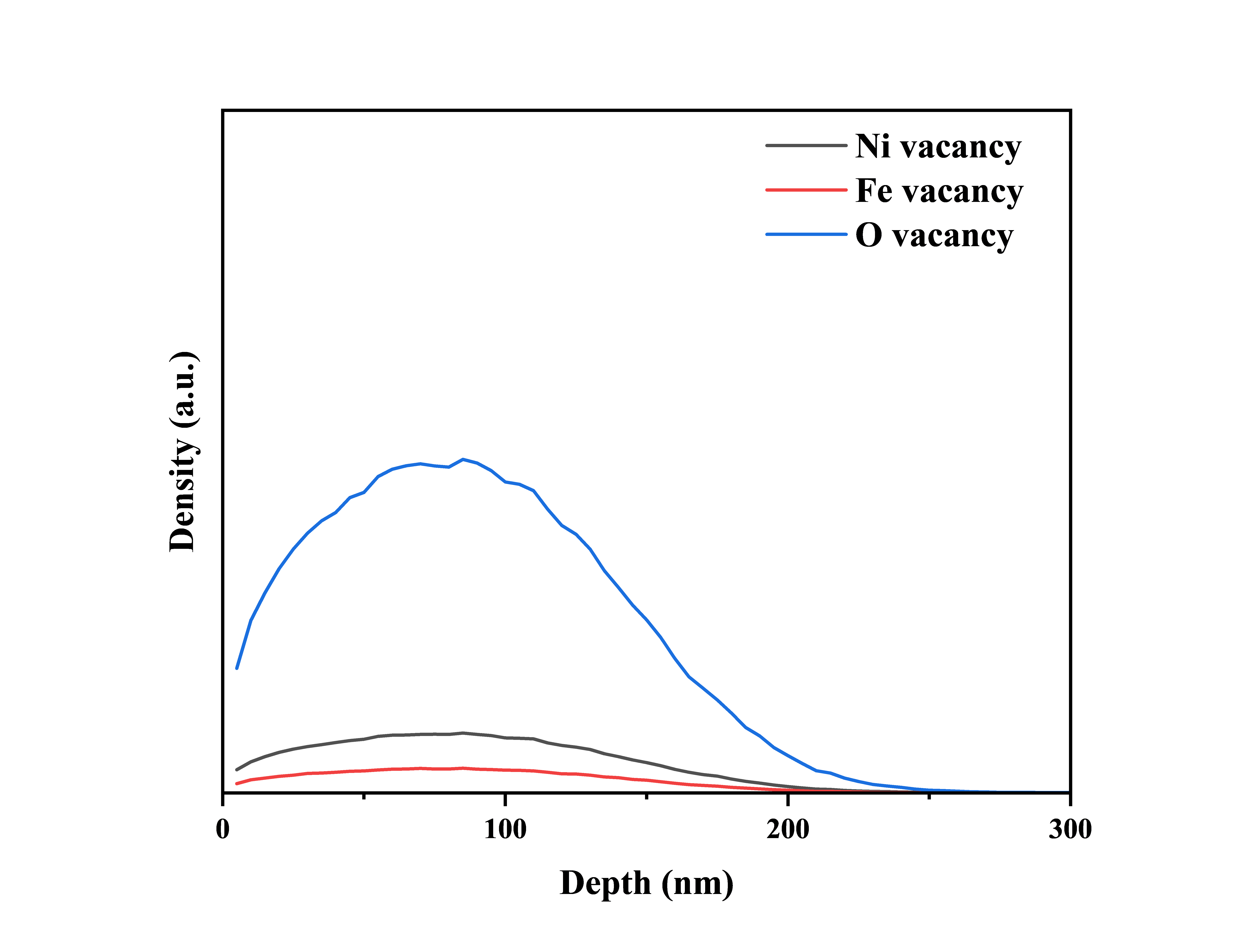


**Figure S11.** SRIM simulations of vacancy concentration distribution of the irradiated samples.


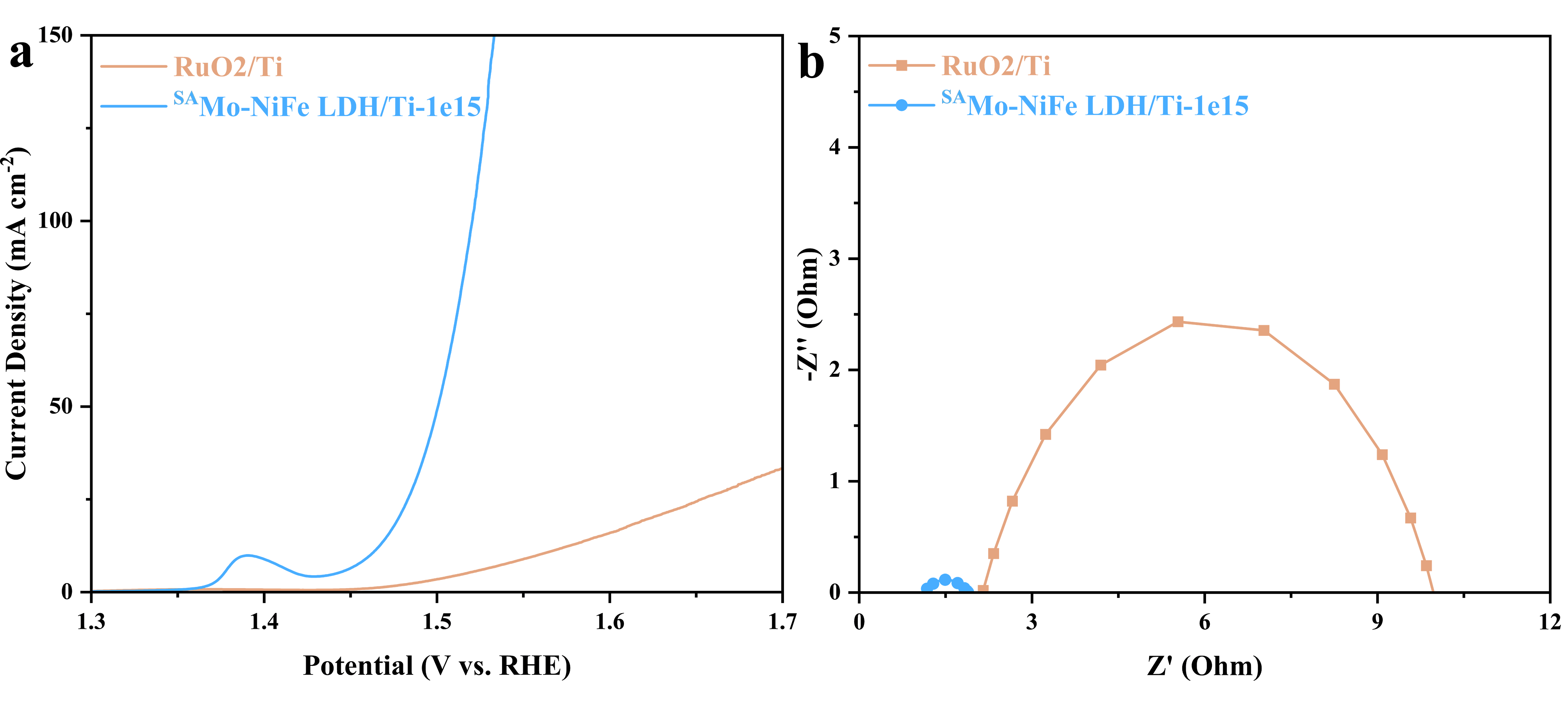


**Figure S12.** a) LSV curves and b)Nyquist diagrams for commercial RuO_2_ and ^SA^Mo-NiFe LDH/Ti-1e15.


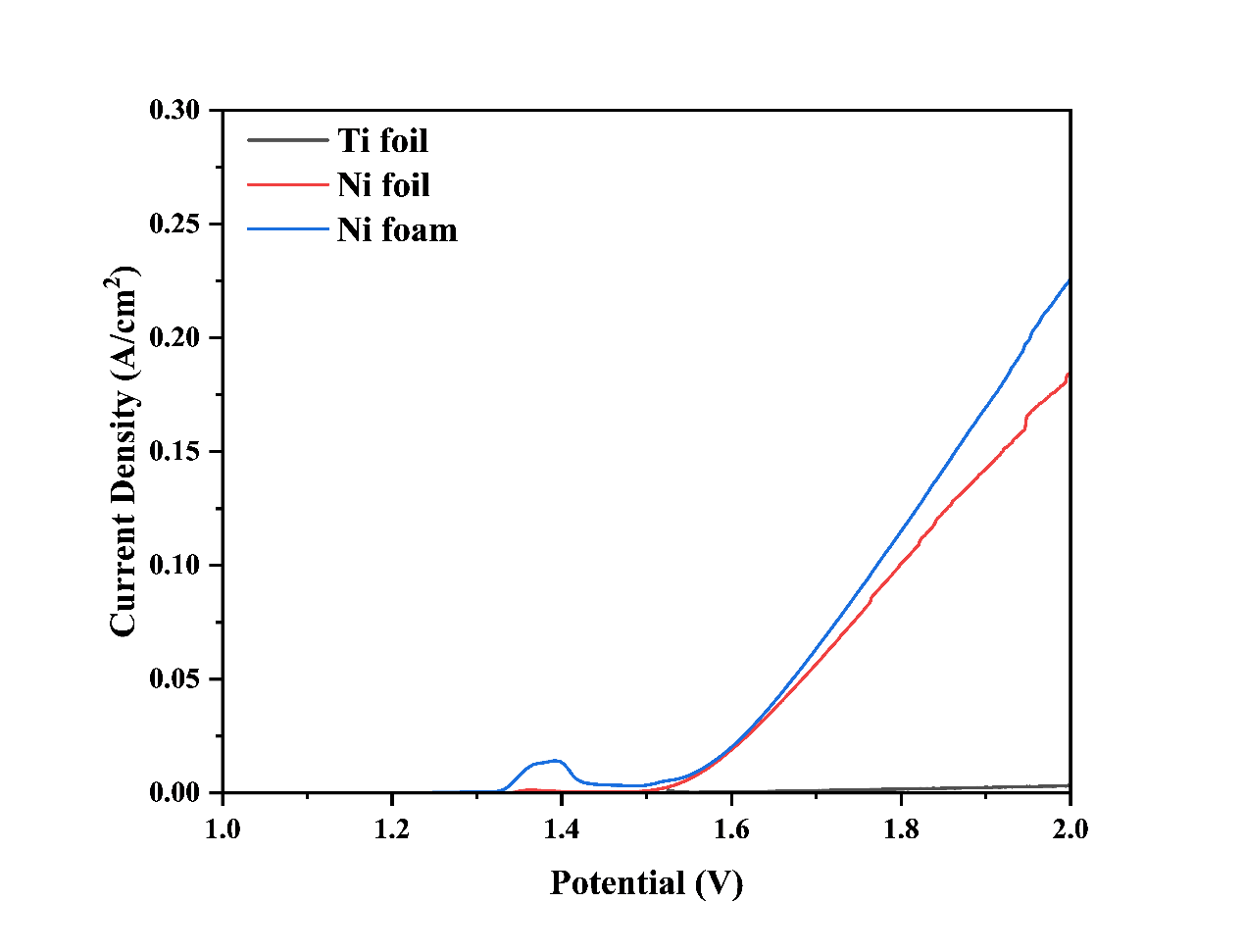


**Figure S13.** LSV curves of Ti foil, Ni foil and Ni foam.


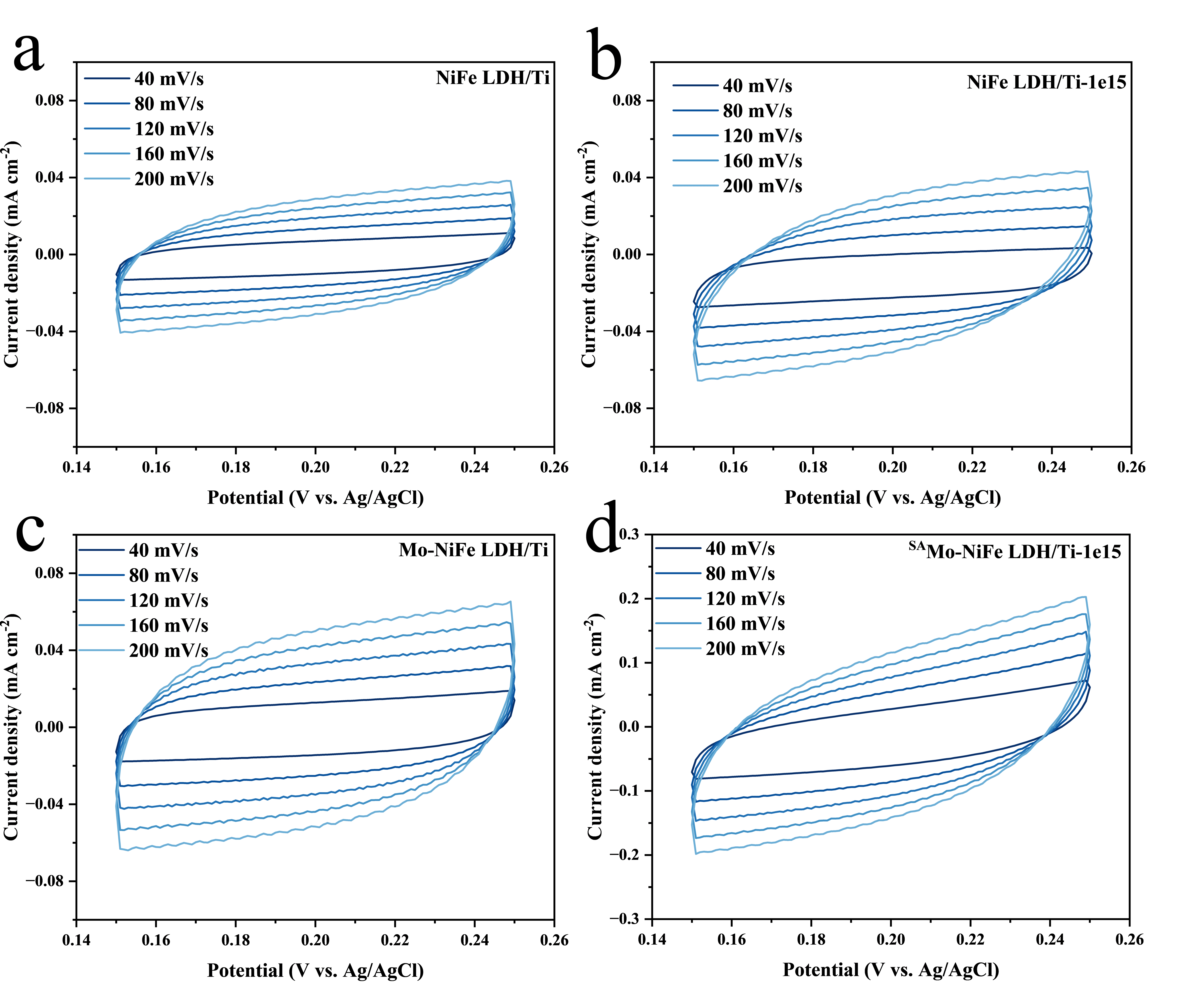


**Figure S14.** a-d) CV diagrams of as-prepared samples at non-Faradaic region.


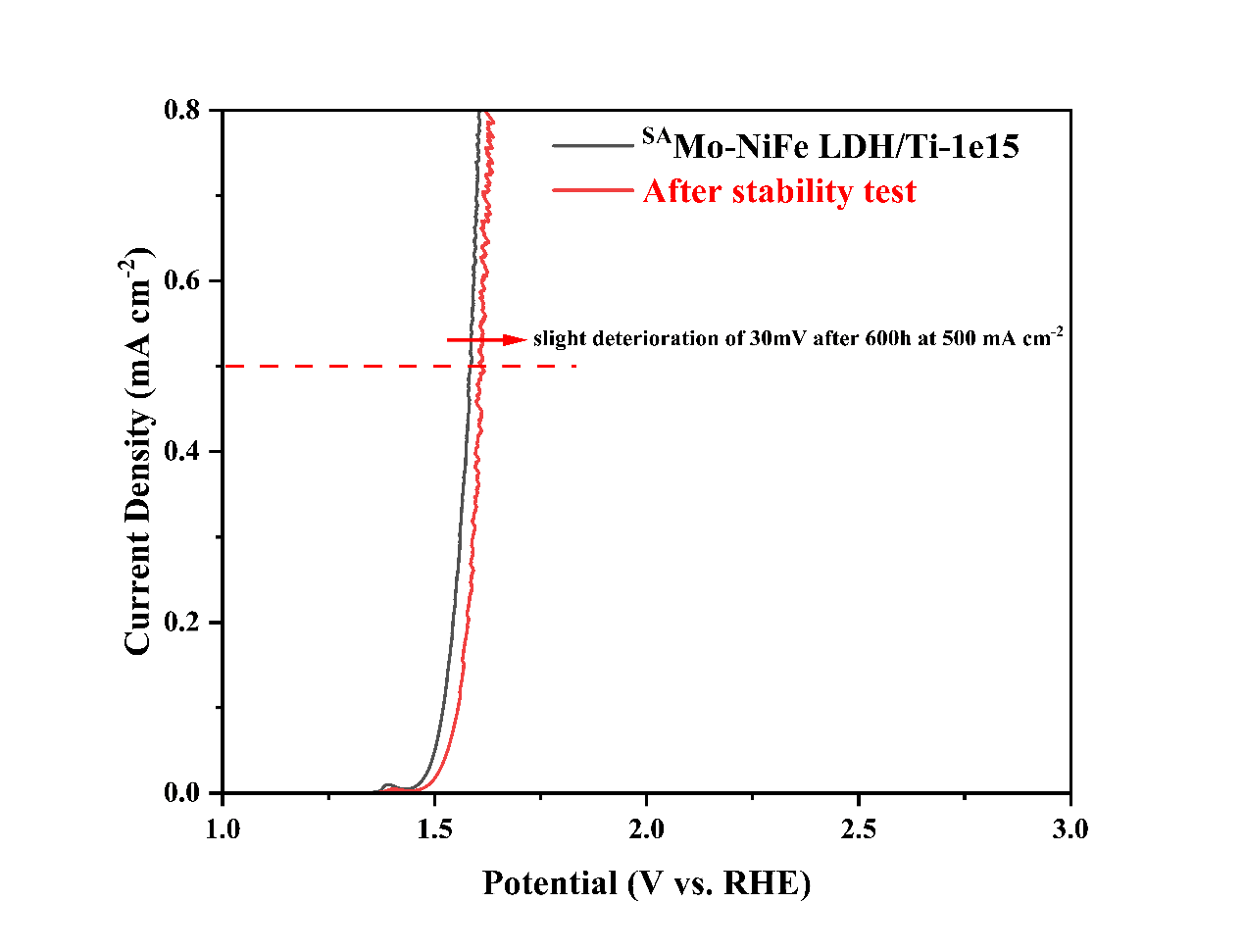


**Figure S15.** LSV curves before and after 600 h stability test for the sample of ^SA^Mo-NiFe LDH/Ti-1e15.


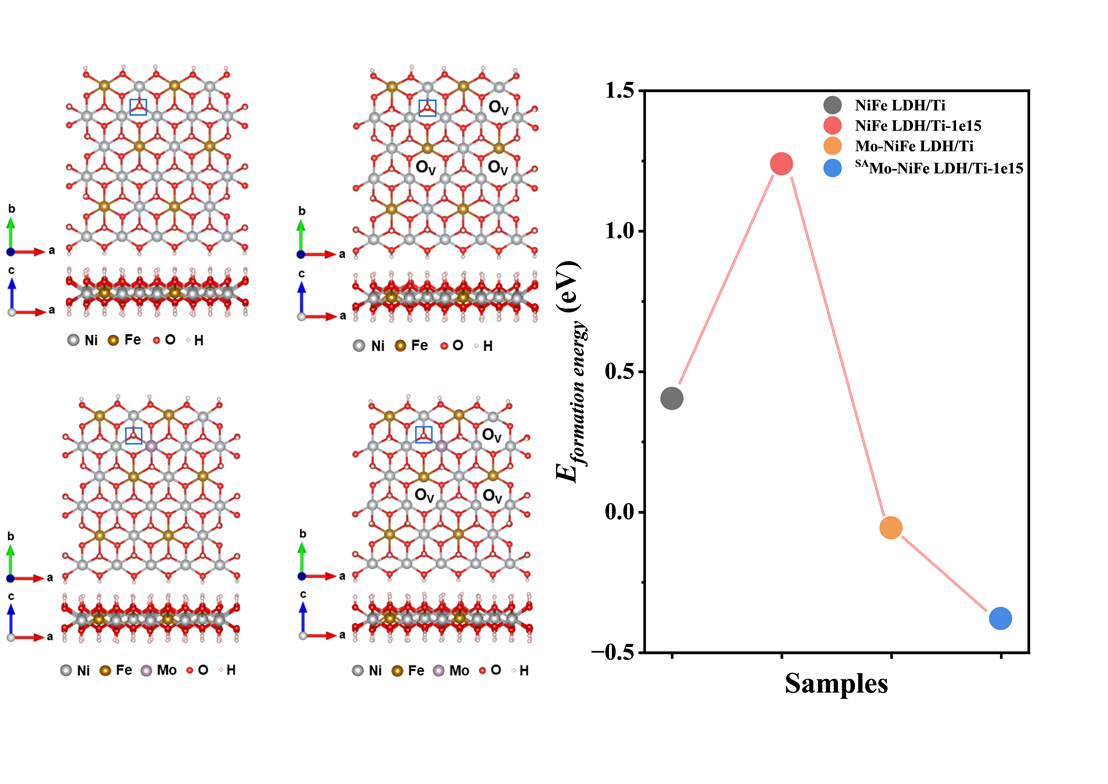


**Figure S16.** DFT calculated formation energy of all samples.


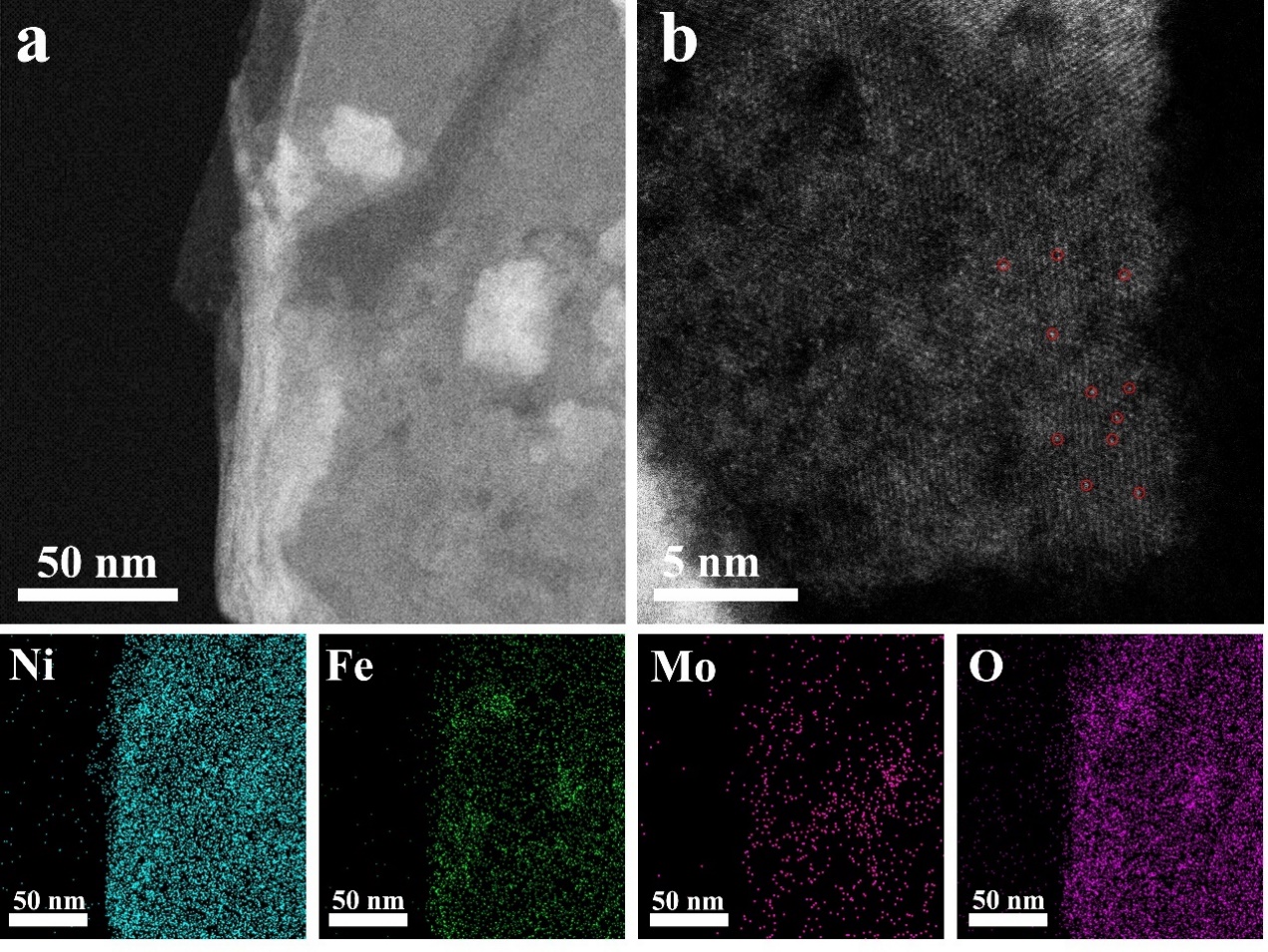


**Figure S17.** Aberration-corrected High resolution STEM images (HAADF-STEM) and corresponding EDS mapping images of ^SA^Mo-NiFe LDH/Ti-1e15 after OER stability test.


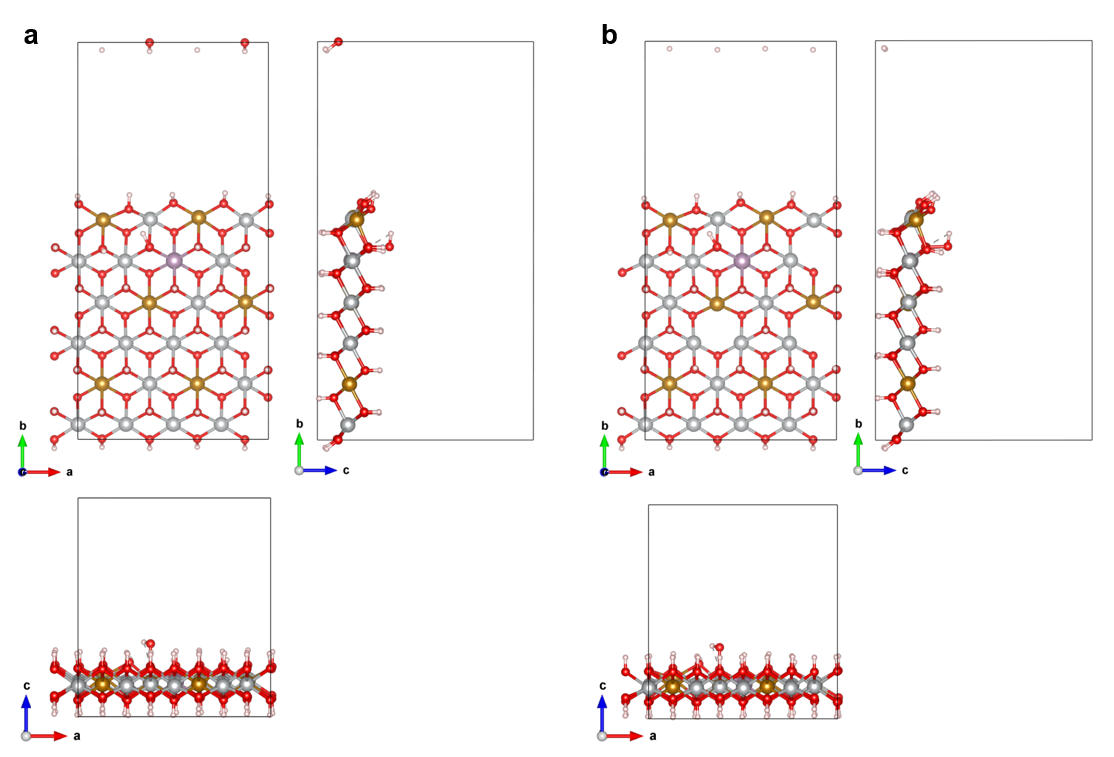


**Figure S18.** Three-dimensional views of the MoNiFe-LDH slab model employed in the DFT calculations: **a** without and **b** with oxygen vacancies.


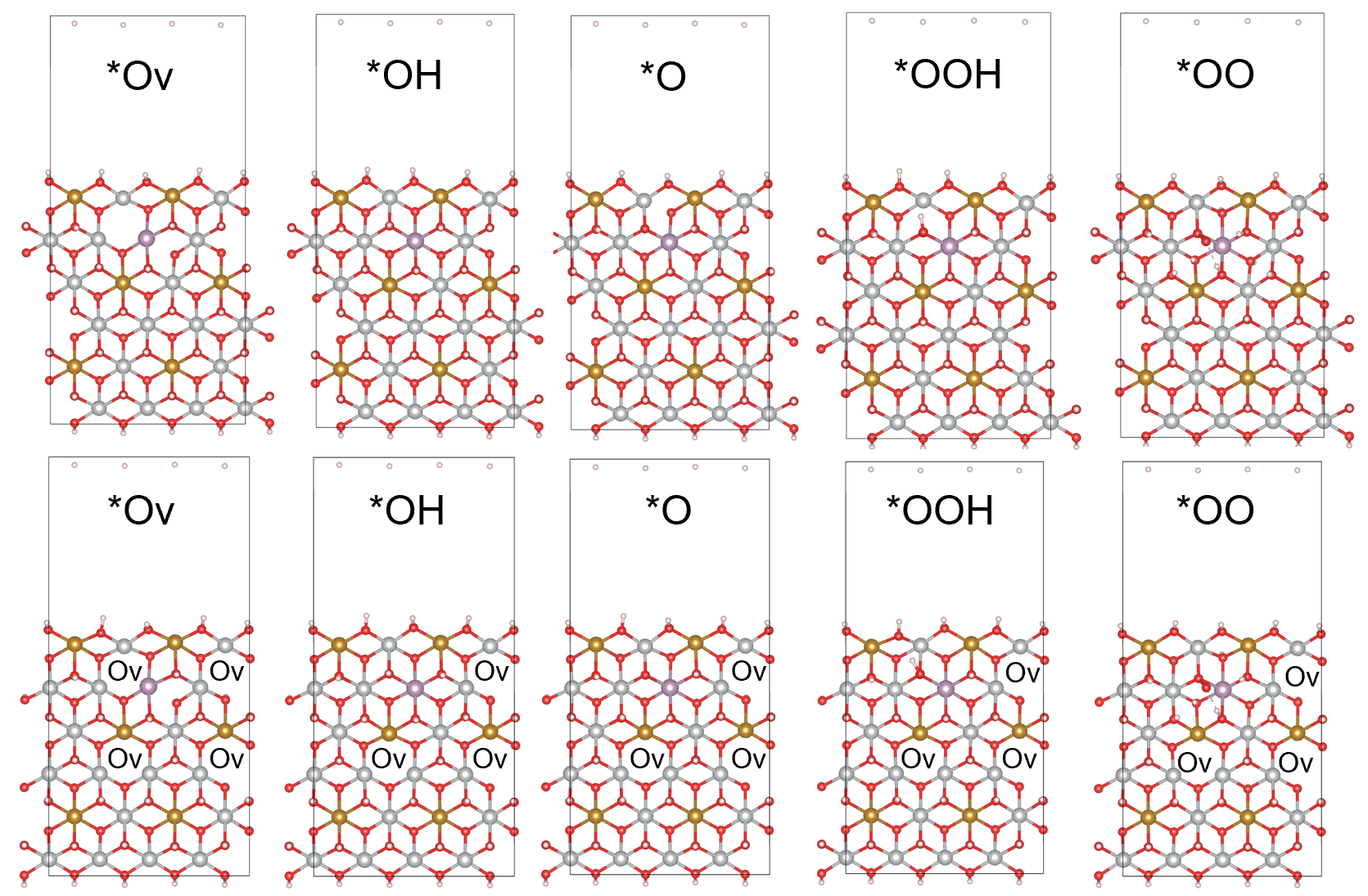


**Figure S19.** The configurations of Mo-NiFe LDH in LOM mechanism.


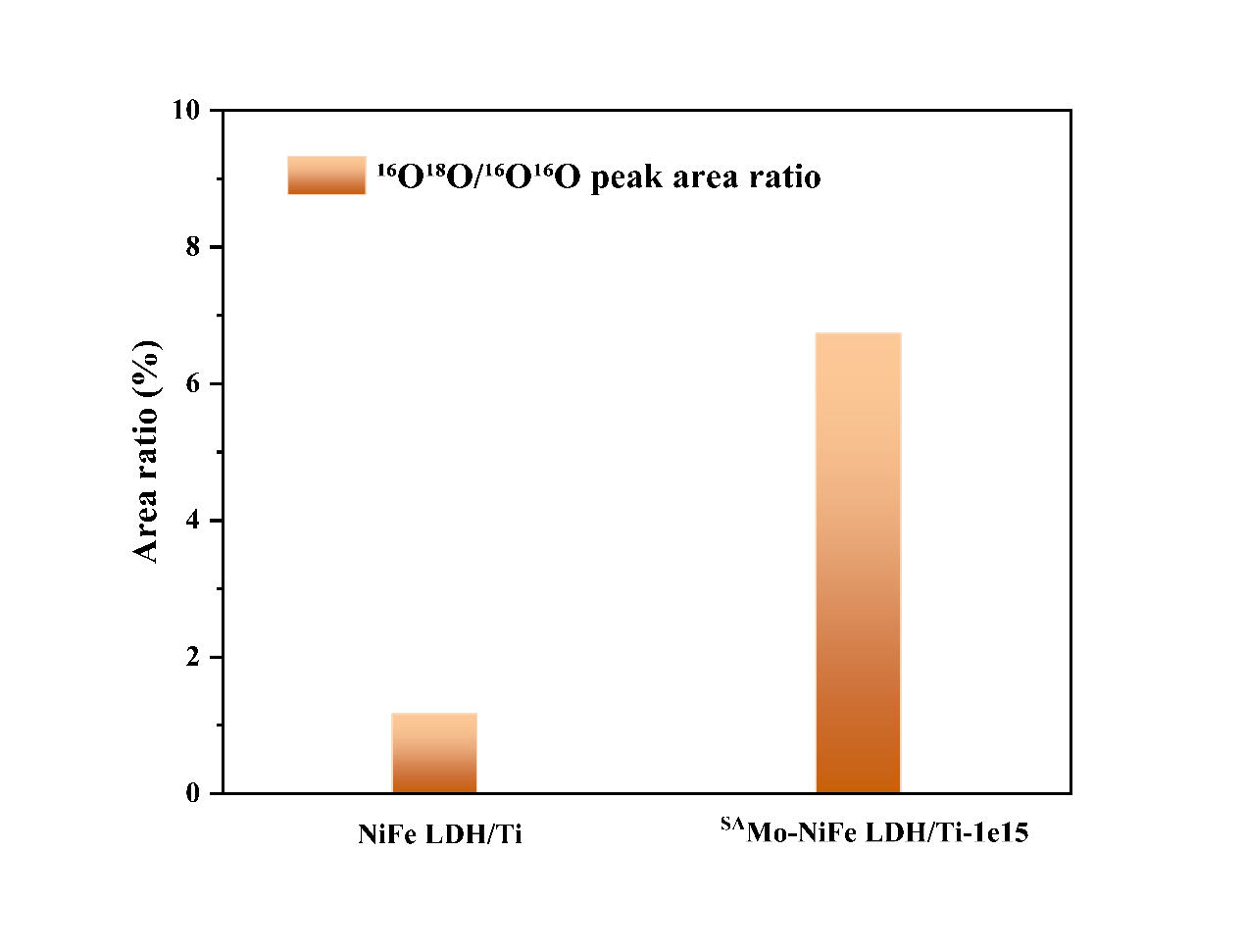


**Figure S20.** Calculated ^16^O^18^O/^16^O^16^O area ratio for samples of NiFe LDH/Ti and ^SA^Mo-NiFe LDH/Ti-1e15.

**Supplementary Tables**

**Table S1.** Elemental analysis of as-synthesized samples through ICP measurements.

| Sample | Ni (wt%) | Fe (wt%) | Mo (wt%) |
| --- | --- | --- | --- |
| NiFe LDH/Ti | 46.32 | 17.06 | - |
| NiFe LDH/Ti-1e15 | 46.52 | 16.56 | - |
| Mo-NiFe LDH/Ti | 44.54 | 16.98 | 2.62 |
| ^SA^Mo-NiFe LDH/Ti-1e15 | 40.52 | 16.56 | 7.35 |

**Table S2**. Comparison of the OER performance of this work with literature data.

| **Catalysts** | **Method** | **△E (mV)** | **Electrolyte** | **η (mV) at j=10 mA cm^-2^** | **Tafel slope**  **(mV dec^-1^)** | **Stability** | **Content (wt%)** | **Ref.** |
| --- | --- | --- | --- | --- | --- | --- | --- | --- |
| **^SA^Mo-NiFe LDH/Ti** | **Ion irradiation strategy** | **226** | **1 M KOH** | **232** | **51.9** | **600 h/500 mA cm^-2^** | **7.4** | **This work** |
| ^SA^Ag-NiCo LDH/CC | Electrodeposition method | 218 | 1 M KOH | 192 | 39 | 500 h/100 mA cm^-2^ | 2.5 | ^[1]^ |
| ^SA^Ru-S-NiFe LDH/CC | Hydrothermal method | 187 | 1 M KOH | 279 | 81.65 | 100 h/10 mA cm^-2^ | 1.9 | ^[2]^ |
| ^SA^Ru-Ni_3_V LDH/NF | Hydrothermal method | 126 | 1 M KOH | 224 | 33 | 240 h/10 mA cm^-2^ | 7.62 | ^[3]^ |
| ^SA^Ru-NiFe LDH/NF | Solution reduction method | 120 | 1 M KOH | 196 | 89.1 | 24 h/10 mA cm^-2^ | 0.52 | ^[4]^ |
| ^SA^Ir-NiCr LDH/CC | Soak method | 118 | 1 M KOH | 232 | 51 | 36 h/20 mA cm^-2^ | 1.59 | ^[5]^ |
| ^SA^Ir-NiFeZn LDH/GCE | Co-precipitation method | 111 | 1 M KOH | 196 | 35 | 2000 cycles | 1.09 | ^[6]^ |
| ^SA^Ru-CoV LDH/NF | Hydrothermal method | 105 | 1 M KOH | 263 | 74.5 | 2000 cycles | 5.23 | ^[7]^ |
| ^SA^Ru-NiFe LDH/CC | Co-precipitation method | 98 | 1 M KOH | 194 | 36 | 100 h/100 mA cm^-2^ | 1.32 | ^[8]^ |
| ^SA^Ru-FeCo LDH/NF | Soak method | 89 | 1 M KOH | 194 | 25 | 1200 h/1000 mA cm^-2^ | 30 | ^[9]^ |
| ^SA^Ce-NiV LDH/NF | Hydrothermal method | 82 | 1 M KOH | 209 | 50.7 | 200 h/50 mA cm^-2^ | 8.07 | ^[10]^ |
| ^SA^Ru-FeCoNi LDH/CC | Self-templating cationexchange method | 74 | 1 M KOH | 205 | 40 | 48 h/10 mA cm^-2^ | 0.67 | ^[11]^ |
| ^SA^NiIr-NiFe LDH/NF | Electrospinning  method | 67 | 1 M KOH | 194 | 32.9 | 120 h/200 mA cm^-2^ | 8.47 | ^[12]^ |
| ^SA^Ru-NiFe LDH/NF | Electrodeposition technique | 64 | 1 M KOH with 7.5 M HMF electrolyte | 243 | 39 | 24 h/10 mA cm^-2^ | 0.52 | ^[13]^ |
| ^SA^Ru-NiFe LDH/NF | Facile electrodeposition and subsequent etching approach | 61 | 1 M KOH | 189 | 31 | 50 h/100 mA cm^-2^ | 1.2 | ^[14]^ |
| ^SA^Rh-NiV LDH/NF | Ethylene glycol-assisted hydrothermal route | 50 | 1 M KOH | 300 | 36 | 41 h/20 mA cm^-2^ | 6.59 | ^[15]^ |
| ^SA^Ru-NiFe LDH/CC | Hydrothermal method | 50 | 1 M KOH | 225 | 78 | 60 h/200 mA cm^-2^ | 0.31 | ^[16]^ |
| ^SA^Pt-NiFe LDH | Thermal reduction | 48 | 1 M KOH | 218 | 37.8 | 12 h/25 mA cm^-2^ | 9.7 | ^[17]^ |
| ^SA^Au-NiFe LDH/Ti | Electrodeposition method | 26 | 1 M KOH | 237 | 36 | 20 h/100 mA cm^-2^ | 0.4 | ^[18]^ |

**Table S3**. Structural parameters extracted from the Mo K-edge EXAFS fitting (^a^S_0_^2^= 0.97). ^a^S_0_^2^ was fixed as 0.97 during EXAFS fitting, based on the known structure of Mo foil. ^b^N is coordination number. ^c^R is the bond length between Mo central atoms and surrounding coordination atoms. ^d^Debye-Waller factor is a measure of thermal and static disorder in absorber-scattering distances. ^e^Goodness of fit. ^f^ΔE_0_ is the difference between the zero kinetic energy value of the sample and that of the theoretical model.

| Sample | Edge | Path | ^b^N | ^c^R (Å) | ^d^σ^2^ (Å^2^) | ^e^R-factor | ^f^△E_0_ (eV) |
| --- | --- | --- | --- | --- | --- | --- | --- |
| Mo foil | Mo | Mo-Mo | 8 | 2.72 | 0.004 | 0.007 | 4.1 |
| ^SA^Mo-NiFe LDH/Ti-1e15 | Mo | Mo-O | 4.0 | 1.79 | 0.005 | 0.02 | 7.5 |
|  |  | Mo-O | 0.9 | 1.88 | 0.004 | 0.07 | 7.5 |

**Table S4**. Fitting results of the EIS plots of samples from Figure 3. R_s_ and R_ct_ represent electrolyte resistance and charge-transfer resistance, respectively.

| **Catalyst** | **R_s_ (Ω)** | **R_ct_ (Ω)** | **CPE-T** | **CPE-P** |
| --- | --- | --- | --- | --- |
| NiFe LDH/Ti | 2.727 | 21.65 | 9.0774E-5 | 0.85262 |
| NiFe LDH/Ti-1e15 | 2.726 | 16.24 | 6.898E-5 | 0.86159 |
| Mo-NiFe LDH/Ti | 2.067 | 4.803 | 1.4488E-4 | 0.91312 |
| ^SA^Mo-NiFe LDH/Ti-1e15 | 1.283 | 0.48621 | 6.3905E-3 | 0.6573 |

**Table S5**. Elemental analysis of Mo-NiFe LDH/Ti and ^SA^Mo-NiFe LDH/Ti-1e14 through ICP measurements.

| Sample | Ni (wt%) | Fe (wt%) | Mo (wt%) |
| --- | --- | --- | --- |
| Mo-NiFe LDH/Ti | 44.54 | 16.98 | 2.62 |
| ^SA^Mo-NiFe LDH/Ti-1e14 | 44.11 | 15.88 | 3.52 |

**References**

1. W. He, R. Zhang, H. Liu, Q. Hao, Y. Li, X. Zheng, C. Liu, J. Zhang, H. L. Xin, "Atomically Dispersed Silver Atoms Embedded in NiCo Layer Double Hydroxide Boost Oxygen Evolution Reaction." *Small* (2023): 2301610, <http://doi.org/10.1002/smll.202301610>.

2. Y. Zhu, J. Wang, G. Weiser, M. Klingenhof, T. Koketsu, S. Liu, Y. Pi, G. Henkelman, X. Shi, J. Li, C. W. Pao, M. H. Yeh, W. H. Huang, P. Strasser, J. Ma, "Ru Single Atoms and Sulfur Anions Dual‐Doped NiFe Layered Double Hydroxides for High‐Current‐Density Alkaline Oxygen Evolution Reaction." *Advanced Energy Materials* (2025): 2500554, <http://doi.org/10.1002/aenm.202500554>.

3. H. Sun, C.-W. Tung, Y. Qiu, W. Zhang, Q. Wang, Z. Li, J. Tang, H.-C. Chen, C. Wang, H. M. Chen, "Atomic Metal–Support Interaction Enables Reconstruction-Free Dual-Site Electrocatalyst." *Journal of the American Chemical Society* (2021): 1174, <http://doi.org/10.1021/jacs.1c08890>.

4. Y. Yang, Q.-N. Yang, Y.-B. Yang, P.-F. Guo, W.-X. Feng, Y. Jia, K. Wang, W.-T. Wang, Z.-H. He, Z.-T. Liu, "Enhancing Water Oxidation of Ru Single Atoms via Oxygen-Coordination Bonding with NiFe Layered Double Hydroxide." *ACS Catalysis* (2023): 2771, <http://doi.org/10.1021/acscatal.2c05624>.

5. S. Biswal, Divya, B. Mishra, D. Pohl, B. Rellinghaus, D. Ghosh, B. P. Tripathi, "Electronic modulation of iridium single atomic sites on NiCr layered double hydroxide for an improved electrocatalytic oxygen evolution reaction." *Journal of Materials Chemistry A* (2024): 2491, <http://doi.org/10.1039/d3ta05848d>.

6. M. Israr, M. Humayun, J. Zhang, K. Shah, X. Tan, C. Chen, Y. Li, "Ir single atoms on NiFeZn-LDH matrix for exceptional oxygen evolution reaction." *Nano Research* (2024): 7039, <http://doi.org/10.1007/s12274-024-6749-4>.

7. K. Zeng, M. Tian, X. Chen, J. Zhang, M. H. Rummeli, P. Strasser, J. Sun, R. Yang, "Strong electronic coupling between single Ru atoms and cobalt-vanadium layered double hydroxide harness efficient water splitting." *Chemical Engineering Journal* (2023): 139151, <http://doi.org/10.1016/j.cej.2022.139151>.

8. X. Duan, P. Li, D. Zhou, S. Wang, H. Liu, Z. Wang, X. Zhang, G. Yang, Z. Zhang, G. Tan, Y. Li, L. Xu, W. Liu, Z. Xing, Y. Kuang, X. Sun, "Stabilizing single-atomic ruthenium by ferrous ion doped NiFe-LDH towards highly efficient and sustained water oxidation." *Chemical Engineering Journal* (2022): 136962, <http://doi.org/10.1016/j.cej.2022.136962>.

9. X. Mu, X. Gu, S. Dai, J. Chen, Y. Cui, Q. Chen, M. Yu, C. Chen, S. Liu, S. Mu, "Breaking the symmetry of single-atom catalysts enables an extremely low energy barrier and high stability for large-current-density water splitting." *Energy & Environmental Science* (2022): 4048, <http://doi.org/10.1039/d2ee01337a>.

10. K. Zeng, M. Chao, M. Tian, J. Yan, M. H. Rummeli, P. Strasser, R. Yang, "Atomically Dispersed Cerium Sites Immobilized on Vanadium Vacancies of Monolayer Nickel‐Vanadium Layered Double Hydroxide: Accelerating Water Splitting Kinetics." *Advanced Functional Materials* (2023): 2308533, <http://doi.org/10.1002/adfm.202308533>.

11. Y. Hu, G. Luo, L. Wang, X. Liu, Y. Qu, Y. Zhou, F. Zhou, Z. Li, Y. Li, T. Yao, C. Xiong, B. Yang, Z. Yu, Y. Wu, "Single Ru Atoms Stabilized by Hybrid Amorphous/Crystalline FeCoNi Layered Double Hydroxide for Ultraefficient Oxygen Evolution." *Advanced Energy Materials* (2020): 2002816, <http://doi.org/10.1002/aenm.202002816>.

12. Y. Hu, T. Shen, Z. Song, Z. Wu, S. Bai, G. Liu, X. Sun, Y. Wang, S. Hu, L. Zheng, Y.-F. Song, "Atomic Modulation of Single Dispersed Ir Species on Self-Supported NiFe Layered Double Hydroxides for Efficient Electrocatalytic Overall Water Splitting." *ACS Catalysis* (2023): 11195, <http://doi.org/10.1021/acscatal.3c02628>.

13. H. Xu, G. Xin, W. Hu, Z. Zhang, C. Si, J. Chen, L. Lu, Y. Peng, X. Li, "Single-atoms Ru/NiFe layered double hydroxide electrocatalyst: Efficient for oxidation of selective oxidation of 5-hydroxymethylfurfural and oxygen evolution reaction." *Applied Catalysis B: Environmental* (2023): 123157, <http://doi.org/10.1016/j.apcatb.2023.123157>.

14. P. Zhai, M. Xia, Y. Wu, G. Zhang, J. Gao, B. Zhang, S. Cao, Y. Zhang, Z. Li, Z. Fan, C. Wang, X. Zhang, J. T. Miller, L. Sun, J. Hou, "Engineering single-atomic ruthenium catalytic sites on defective nickel-iron layered double hydroxide for overall water splitting." *Nature Communications* (2021): 4587, <http://doi.org/10.1038/s41467-021-24828-9>.

15. H. Sun, L. Li, H.-C. Chen, D. Duan, M. Humayun, Y. Qiu, X. Zhang, X. Ao, Y. Wu, Y. Pang, K. Huo, C. Wang, Y. Xiong, "Highly efficient overall urea electrolysis via single-atomically active centers on layered double hydroxide." *Science Bulletin* (2022): 1763, <http://doi.org/10.1016/j.scib.2022.08.008>.

16. H. T. Dao, V. H. Hoa, S. Sidra, M. Mai, M. Zharnikov, D. H. Kim, "Dual efficiency enhancement in overall water splitting with defect-rich and Ru atom-doped NiFe LDH nanosheets on NiCo2O4 nanowires." *Chemical Engineering Journal* (2024): 150054, <http://doi.org/10.1016/j.cej.2024.150054>.

17. W. Chen, B. Wu, Y. Wang, W. Zhou, Y. Li, T. Liu, C. Xie, L. Xu, S. Du, M. Song, D. Wang, Y. liu, Y. Li, J. Liu, Y. Zou, R. Chen, C. Chen, J. Zheng, Y. Li, J. Chen, S. Wang, "Deciphering the alternating synergy between interlayer Pt single-atom and NiFe layered double hydroxide for overall water splitting." *Energy & Environmental Science* (2021): 6428, <http://doi.org/10.1039/d1ee01395e>.

18. J. Zhang, J. Liu, L. Xi, Y. Yu, N. Chen, S. Sun, W. Wang, K. M. Lange, B. Zhang, "Single-Atom Au/NiFe Layered Double Hydroxide Electrocatalyst: Probing the Origin of Activity for Oxygen Evolution Reaction." *Journal of the American Chemical Society* (2018): 3876, <http://doi.org/10.1021/jacs.8b00752>.
